# A dopaminergic mechanism of antipsychotic drug efficacy, failure, and failure reversal: the role of the dopamine transporter

**DOI:** 10.1038/s41380-018-0114-5

**Published:** 2018-07-23

**Authors:** Davide Amato, Fabio Canneva, Paul Cumming, Simone Maschauer, Dominik Groos, Jana Katharina Dahlmanns, Teja W. Grömer, Lisa Chiofalo, Marc Dahlmanns, Fang Zheng, Johannes Kornhuber, Olaf Prante, Christian Alzheimer, Stephan von Hörsten, Christian P. Müller

**Affiliations:** 1 Department of Psychiatry and Psychotherapy, University Clinic, Friedrich-Alexander-University Erlangen-Nürnberg, Erlangen, Germany; 2 Department of Neuroscience, Medical University of South Carolina, Charleston, SC USA; 3 Department of Experimental Therapy, Preclinical Experimental Center, Friedrich-Alexander-University Erlangen-Nürnberg, Erlangen, Germany; 4 Department of Nuclear Medicine, Molecular Imaging and Radiochemistry, Friedrich-Alexander-University Erlangen-Nürnberg, Erlangen, Germany; 5 School of Psychology and Counselling and IHBI, Queensland University of Technology, and QIMR Berghofer Medical Research Institute, Brisbane, Australia; 6 Institute of Physiology and Pathophysiology, Friedrich-Alexander-University Erlangen-Nürnberg, Erlangen, Germany

**Keywords:** Schizophrenia, Predictive markers, Schizophrenia, Predictive markers

## Abstract

Antipsychotic drugs are effective interventions in schizophrenia. However, the efficacy of these agents often decreases over time, which leads to treatment failure and symptom recurrence. We report that antipsychotic efficacy in rat models declines in concert with extracellular striatal dopamine levels rather than insufficient dopamine D2 receptor occupancy. Antipsychotic efficacy was associated with a suppression of dopamine transporter activity, which was reversed during failure. Antipsychotic failure coincided with reduced dopamine neuron firing, which was not observed during antipsychotic efficacy. Synaptic field responses in dopamine target areas declined during antipsychotic efficacy and showed potentiation during failure. Antipsychotics blocked synaptic vesicle release during efficacy but enhanced this release during failure. We found that the pharmacological inhibition of the dopamine transporter rescued antipsychotic drug treatment outcomes, supporting the hypothesis that the dopamine transporter is a main target of antipsychotic drugs and predicting that dopamine transporter blockers may be an adjunct treatment to reverse antipsychotic treatment failure.

## Introduction

Antipsychotic drugs (APDs) are a mainstay in the treatment of schizophrenia. The available APDs exhibit a large spectrum of mechanisms and act on receptors of diverse biogenic monoamine neurotransmitters [1]. However, D_2_ receptor blockade is a common property of effective APD medications [2]. It is often suggested that the maximal clinical response is achieved when APDs block ~65–75% of D_2_ receptors and is negligible when D_2_ receptor occupancy is below this therapeutic window [3, 4]. However, a growing body of evidence appears to cast doubt on the causal effect of this mechanism [5]. Although antipsychotic therapeutic efficacy may appear positively associated with receptor blockade at the beginning of the treatment, APDs exhibit declining therapeutic efficacy with long-term treatment even though their receptor occupancy remains fairly stable. Clinical Antipsychotic Trials of Intervention Effectiveness (CATIE) studies define the incremental lack of antipsychotic efficacy as one of the main factors of drug discontinuation, which was observed within 18 months after treatment initiation in 75% of patients [6–8]. Related to this acquired pharmacological resistance, ~20% of all schizophrenia patients will never respond to APDs, even if a consistent D_2_ receptor occupancy is maintained at the start of the treatment within the therapeutic window [9–11]. Additionally, clinical evidence shows that there is indeed a relationship between early response/no-response and long-term antipsychotic outcomes [12]. While the heterogeneous neurobiology underlying schizophrenia symptoms could lead to multiple antipsychotic responses, the pharmacological mechanisms beyond D_2_ receptors regulating APD treatment efficacy and failure still await clarification. In this study we bridge this gap by modelling APD treatment efficacy and failure in psychopathologically naïve animals. Specifically, we aimed to recapitulate APD-driven neuroadaptations that can contribute to core clinical issues including long-term lack of efficacy, relapse and APD treatment-resistance. To understand the neurobiology of APDs efficacy and failure, we probed the underlying neurochemical and neurophysiological mechanisms and developed a rational pharmacological intervention to ameliorate the loss of APD effects.

## Materials and Methods

### Animals

Male Sprague-Dawley rats (Charles River Laboratories, Germany) weighing 250–300 g were used for most of the studies. Male C57Bl/6 6-week old mice (Charles River Laboratories, Germany) were used for the electrophysiology studies only. All experiments were performed in accordance with the Animal Protection Law of the Federal Republic of Germany and the European Communities Council Directive of 24 November 1986 (86/609/EEC), and local authorities approved all study protocols.

### Drugs and treatment methods

We used clinical equivalent doses of haloperidol (HAL) and olanzapine (OLA), as described in the supplementary material based on previous work [13, 14]. d-amphetamine (AMPH) and the dopamine transporter (DAT) blocker GBR 12909 were administered intraperitoneally (i.p.). GBR 12909 was also administered locally into the CPu (left and right sides) at 20 µg in 1 µl volume per side, according to previously described procedures [15, 16]. A challenge of 100 mM potassium chloride (K^+^) was delivered for 80 min to the medial prefrontal cortex (mPFC), caudate-putamen (CPu), and nucleus accumbens (NAcc) via reverse dialysis, as previously described [17]. Full methodological procedures are described in the supplementary material.

### Behavior

Locomotion, acoustic startle reflex (ASR) and pre-pulse inhibition (PPI) were recorded prior to and after either vehicle (veh) or AMPH (3 mg/kg) injections or in response to a tail-pinch (TP). We monitored the ability of HAL (2 and 14 days) and OLA (2, 6, 14, and 21 days) to inhibit the AMPH-induced behavioral disruptions. Additional methodological details are described in the supplementary material.

### Microdialysis and behavior

Microdialysis studies were coupled with behavioral analyses according to previous protocols [13, 14]. Particularly, we investigated the efficacy of HAL (6 and 14 day) and OLA (2, 6, and 21 days) to inhibit dopamine release and locomotion induced by a TP. We also measured the effect of systemic and intrastriatal (according to previous procedures [15, 16]) GBR12909 injections in the reversal of HAL treatment failure. Further details are described in the supplementary material.

### Western blot analysis and PCR studies

DAT, serotonin (SERT), and noradrenaline transporters (NET) and tyrosine hydroxylase (TH) protein expression levels were measured using standard Western blot analysis in brain samples from rats treated with chronic veh or HAL (see Methods section in supplementary material and Figure S4). We also measured DAT expression levels after OLA treatment (supplementary Figure S5). Brain tissue was acquired at 2, 6, and 14 days of treatment, and the regions of interest (CPu, NAcc, and PFC) were rapidly dissected on ice, according to previous methods [18]. We also determined the nigrostriatal and mesolimbic DAT mRNA expression after veh or HAL treatment. The DAT mRNA expression levels were quantified in freshly dissected portions of striatum using a standard reverse transcriptase-coupled quantitative real-time PCR (qRT-PCR) procedure and the primer pairs DAT-for-AGCTACCATGCCCTATGTGG and DAT-rev-ATCAGCACTCCAAACCCAAC (see supplementary material for details).

### MicroPET brain study

We determined striatal dopamine D_2/3_ receptor and DAT availability according to previously described methods using [^18^F]fallypride [19] and [^18^F]FP-CMT [20], respectively (see details in the supplementary material). To be conform with the literature we will use “D_2_” instead of “D_2/3_” receptors. We measured striatal dopamine D_2_ receptor availability after 14 days of treatment with veh or HAL. Image reconstruction was performed as described previously [20, 21], and parametric maps of the binding potential (BP_ND_) were calculated using the simplified reference tissue method (SRTM) [22]. We also measured the DAT density availability at baseline and on day 14 of HAL treatment (follow-up). Pairs of BP_ND_ maps were calculated relative to the cerebellum TAC, as documented in our previous characterization of this DAT ligand [20, 23].

### Brain slice electrophysiology study

Veh- or HAL-treated C57BL6 mice were anaesthetized with sevoflurane, and brain slices (250–300 µm) containing the midbrain or dorsal striatum were prepared. Whole-cell recordings of visualized neurons in the substantia nigra pars compacta (SNc) were performed as previously described [17] (see details in the supplementary material).

### Neurophotonic study

Primary hippocampal neurons were cultured and transfected with synapto-pHIuorin (spH). Synaptic vesicle exocytosis in HAL and veh treatment groups was recorded using a fluorescence microscope as previously described [17]. Further details are described in the supplementary material.

### Statistical analyses

Data analyses and a summary of the results are reported in the supplementary material.

## Results

### Behavioral expression of antipsychotic treatment efficacy and failure

**Fig. 1:**
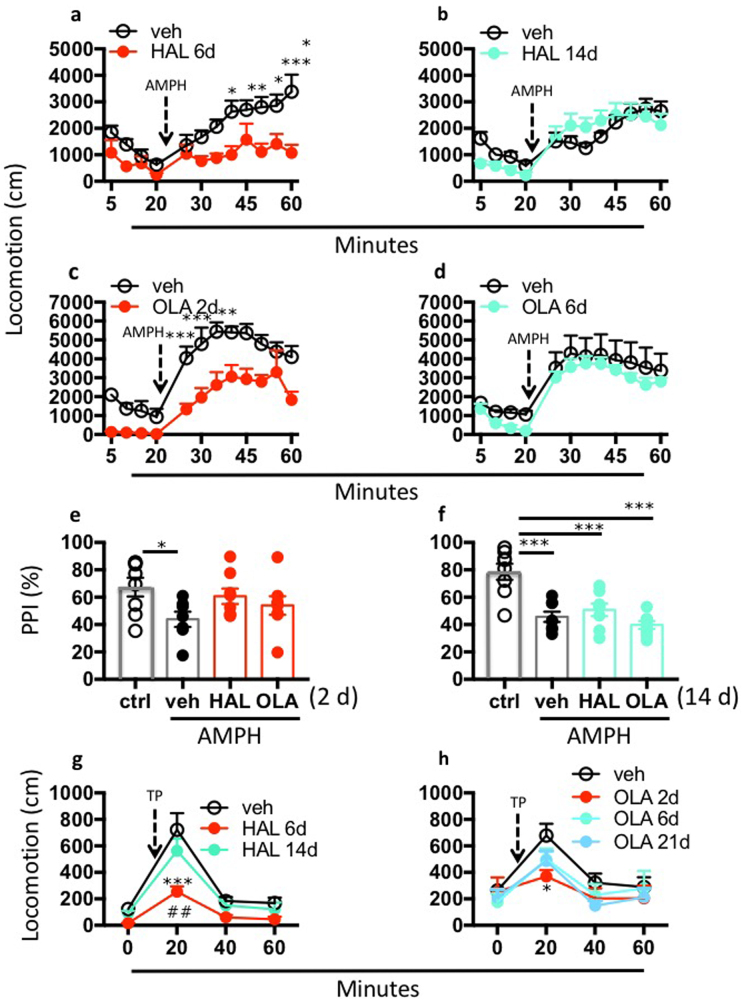
Behavioral expression of antipsychotic treatment efficacy and failure. Short- and long-term efficacy of haloperidol (HAL) and olanzapine (OLA) in blocking the effects of amphetamine (AMPH) and tail pinch (TP) on behavioral activity. **a, b** HAL (0.5 mg/kg/d) treatment for 6 days reduced AMPH-induced (2 mg/kg) hyperlocomotion (*P* < 0.01, main effect), but no longer after 14 days treatment. *N* = 7/group. **c, d** OLA (10 mg/kg/d) treatment for 2 days reduced AMPH-induced (2 mg/kg) hyperlocomotion (*P* < 0.01, main effect), but no longer after 6 days treatments. *N* = 5/group. **e, f** HAL (0.5 mg/kg/d) or OLA (10 mg/kg/d) prevented the PPI reduction induced by AMPH (3 mg/kg, vehicle (veh) vs. control (ctrl.), *P* < 0.05) after 2 days, but no longer after 14 days treatment (HAL: *P* = 0.0001; OLA: *P* < 0.0001). **g** HAL (0.5 mg/kg/d) treatment for 6 days reduced TP (20 min)-induced hyperlocomotion compared with veh (*P* < 0.0001), but no longer after 14 days treatment. *N* = 7–17/group. **h** OLA (10 mg/kg/d) treatment for 2 days reduced TP (20 min)-induced hyperlocomotion compared with veh (*P* < 0.05), but no longer after the continuous treatment. *N* = 5–20/group. All data are means ± S.E.M *P < 0.05, **P < 0.01, ***P < 0.001, ****P < 0.0001. Statistical significance represents post hoc comparison when not specified

We compared the efficacy of short- (2–6 days) and long-term (6–21 days) treatment with HAL (0.5 mg/kg/d) or OLA (10 mg/kg/d) in the inhibition of AMPH-induced hyperlocomotion and reversal of sensorimotor gating deficits in the PPI of the startle reflex test in rats. To prevent potential experimental biases related to the use of AMPH, we also measured the time-course of APD efficacy in a non-pharmacological model based on the hyperlocomotion induced by a TP. TP stimulates locomotor activity and promotes striatal dopamine release [24], which mimics the effects of AMPH. We found that short-term, but not chronic, HAL and OLA treatments at clinically relevant doses reversed the PPI deficit and blocked AMPH- and TP-induced hyperlocomotion, thus showing the efficacy of APD after short-term treatment and its declining efficacy with long-term treatment in all tests in rats (Fig. 1a–h). These results suggest that the loss of efficacy is a robust consequence of long-term APD treatment in animal models. No clear signs of abnormal motor behavior were observed during daily inspection throughout treatment periods.

### D_2_ receptor binding and extracellular dopamine levels during antipsychotic treatment failure

**Fig. 2:**
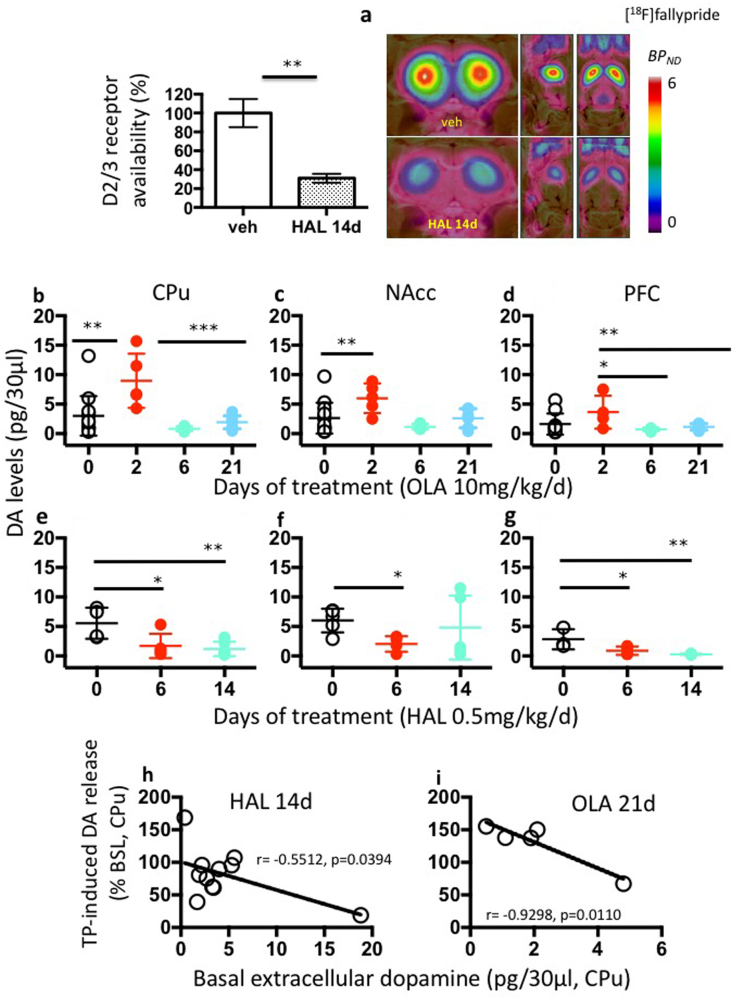
Dopamine D_2_ receptor binding and extracellular dopamine levels during antipsychotic treatment failure. **a** Mean parametric maps in the coronal, left sagittal, and horizontal planes of the binding potential (BP_ND_) of the D_2_ receptor radioligand [^18^F]fallypride determined by PET imaging in animals treated with vehicle (upper row; veh) or with haloperidol (HAL) for 14 days (lower row, HAL 14d). The images represent the mean of four separate animals in each group. The mean D_2/3_ receptor occupancy under HAL treatment was 69 ± 15% (*n* = 4, *P* < 0.001, unpaired *t*-test Fig. 2a), as calculated relative to the mean BP_ND_ in four vehicle control animals (occupancy = [BP(veh)-BP(HAL 14d)]/BP(veh) × 100%). The parametric maps were spatially normalized to a standard histological atlas of rat brain. **b–g** Changes in extracellular dopamine in caudate-putamen (CPu), nucleus accumbens (NAcc), and pre-frontal cortex (PFC) after continuous treatment with olanzapine (OLA) or HAL. **b** OLA increased extracellular dopamine levels in the CPu after 2 days treatment compared to control (day 0) (*P* = 0.001) and to all other treatment periods (*P* < 0.001). **c** OLA increased extracellular dopamine levels in the NAcc after 2 days treatment compared to control (day 0) (*P* < 0.01). **d** In PFC, 2 days OLA treatment increased dopamine levels compared with groups treated for 6 and 21 days (*P* < 0.05), but not compared with control (day 0). *N* = 4–14/group. **e** HAL decreased dopamine levels in CPu after 6 days (*P* < 0.05) and after 14 days (*P* < 0.01) compared with control (day 0). *N* = 4–6/group. **f** HAL decreased dopamine levels in PFC after 6 days (*P* < 0.05) and after 14 days (*P* < 0.01) compared with control (day 0). *N* = 3–4/group. **g** HAL decreased dopamine levels in NAcc after 6 days (*P* < 0.01) as detected post doc using a *t*-test, but not after 14 days. *N* = 4–5/group. Data are means ± S.D. *P < 0.05, **P < 0.01, ***P < 0.001. Statistical significance represents post hoc comparison when not specified. **h–i** Relationship between extracellular dopamine levels at the baseline in the CPu (*X*-axis) and dopamine release in the CPu *(Y*-axis) in response to tail-pinch (TP), expressed in percentage change of the baseline. **h** The reduced dopamine levels provoked by 14 days HAL treatment correlated with the dopamine release stimulated by TP (pearson *r* = -0.5512, *P* < 0.05). *N* = 11/group. **i** The reduced dopamine levels driven by 21 days treatment with OLA correlated with the dopamine output stimulated by TP (pearson *r* = -0.9298, *P* < 0.05). *N* = 5/group

The occurrence of relapse during APD treatment of schizophrenia is attributed to an excessive potentiation of dopaminergic neurotransmission (dopamine supersensitivity) [25–28]. We hypothesized that concerted changes in receptor density [25, 29–31] and/or sensitivity [27, 32] may reduce APD occupancy at D_2_ receptors to below the therapeutic threshold. We measured the in vivo availability of striatal D_2_ receptors using positron emission tomography (PET) with [^18^F]fallypride in rats undergoing 14 days HAL treatment, i.e., during the HAL loss of efficacy, to investigate this hypothesis. Animals were not challenged with AMPH or TP and the decreased efficacy of HAL in this group of animals was based on the results of the behavioral testing of independent subjects described above. We found that ~69% of striatal D_2_ receptors were occupied by HAL (Fig. 2a), which indicates that the loss of HAL efficacy observed in the behavioral tests might occur despite constant and significant D_2_ receptor occupancy by HAL.

We also found that extracellular dopamine levels in rat CPu decreased with prolonged HAL and OLA treatments (Fig. 2b–g) and that individual dopamine levels correlated with the dopamine response to a TP stimulus (Fig. 2h, i). These results demonstrate that APD efficacy in the rat declines in concert with extracellular dopamine levels but not insufficient D2 receptor occupancy by APDs.

### Dopamine synthesis, release and clearance capacity during antipsychotic treatment efficacy and failure

Extracellular dopamine concentrations are controlled via physiological mechanisms that establish a balance between release and re-uptake [33]. We investigated adaptations in the mechanisms of dopamine synthesis, release and clearance in relation to APD treatment efficacy and failure. We measured TH and DAT expression using Western blot analysis during HAL and OLA treatment. During treatment failure, we observed increased TH expression in the CPu (Supplementary Figure 1a, d) but not in the NAcc or PFC compared to vehicle (Supplementary Figure 1b, c, e, f). Also, APD did not alter the group mean of DAT expression in the CPu, NAcc, or PFC (Supplementary Figure 2–3a, c, e). This result may erroneously suggest an absence of effect at this target. However, we detected strong inter-individual APD treatment-induced changes in DAT expression. Therefore, we described this variability by applying a standard procedure to calculate the mean absolute deviation (MAD) [34] using the following formula: individual data value – group mean value [*x* − (Σ*x*/*n*)]. The value of each individual DAT expression at a specific treatment point (i.e., 2, 6, 14, or 21 days) was subtracted from the group mean at the same treatment point.

**Fig. 3:**
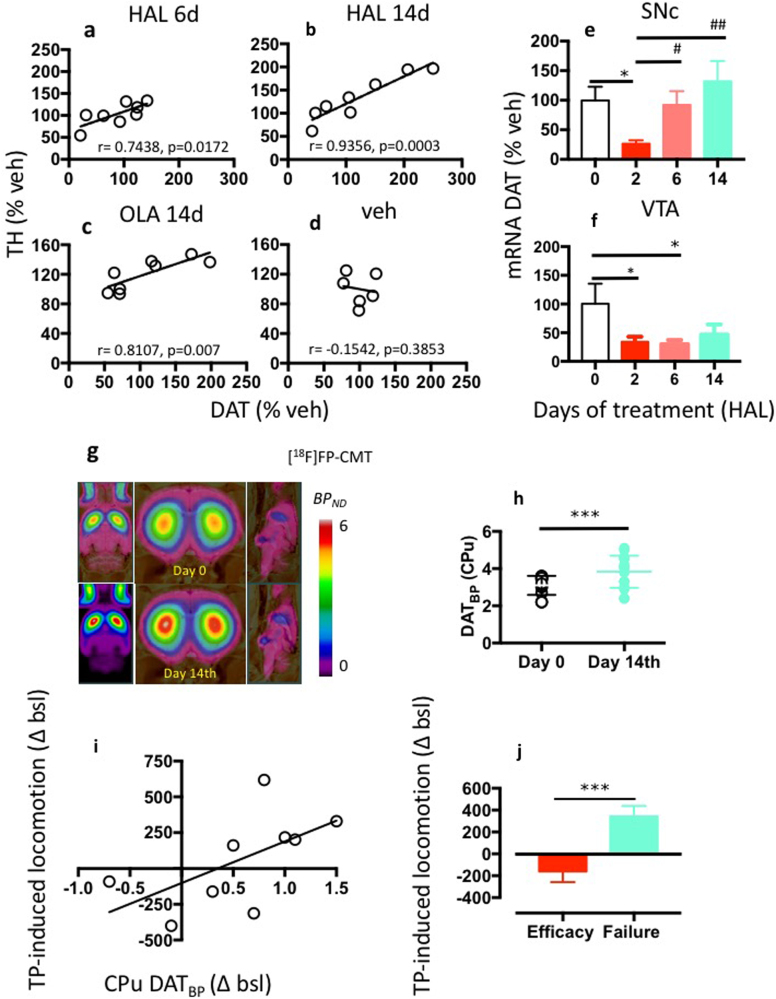
Dopamine synthesis, release and clearance capacity during antipsychotic treatment failure. **a, b** The expression of dopamine transporter (DAT) and tyrosine hydroxylase (TH) is coregulated after 6 days (Pearson *r* = 0.7438, *P* < 0.05) and 14 days (Pearson *r* = 0.9356 *P* < 0.001) HAL treatment. *N* = 8/group. **c** The expression of DAT and TH is coregulated by 21 days olanzapine (OLA) treatment (Pearson *r* = 0.8107, *P* < 0.01), but not by the treatment with vehicle (veh) (**d**). *N* = 8/group. **e,f** Changes in DAT mRNA expression in the substantia nigra (SNc) and in the ventral tegmental area (VTA) after continuous treatment with haloperidol (HAL). HAL treatment for 2 days decreased DAT mRNA expression in the SNc compared with veh (*P* < 0.05, main effect) and to 6 (*P* < 0.05) and 14 (*P* < 0.01) days HAL. *N* = 7–8/group. Data are means ± S.E.M (*P < 0.05 – vs. veh, ^##^*P* < 0.01 – vs. 2d HAL treatment). **f** HAL treatment for 2 and 6 days decreased DAT mRNA expression in the VTA compared to veh (*p* < 0.05). This inhibitory effect vanished after 14 days. *N* = 7–8/group. All data are means ± S.E.M (*P < 0.05, **P < 0.01, ***P < 0.001). **g** DAT density (expressed as mean parametric maps of binding potential (BP_ND_)) in animals scanned first at baseline and after 14 days HAL treatment, as determined by PET imaging with [^18^F]FP-CMT. **h** HAL treatment for 14 days increased the mean group of DAT_BP_ in the caudate-putamen (CPu) compared with baseline (*P* < 0.001). *N* = 16/group. Data are means ± S.D. **i** Relationship between DAT_BP_ changes (calculated as delta between follow-up and baseline) in the CPu and hyperlocomotion response to tail pinch (TP) (calculated as delta between stimulation and baseline) (pearson *r* = 0.7167, *P* < 0.05). *N* = 9/group. Data are means ± S.D. **j** Animals with a DAT_BP_ < 0.8 were experiencing treatment efficacy compared with the subgroup with a DAT_BP_ > 0.8 who were instead prone to antipsychotic treatment failure (*P* < 0.01). *N* = 9/group. Data are means ± S.E.M. (*P < 0.05, **P < 0.01, ***P < 0.001). Statistical significance represents post hoc comparison when not specified

We found inter-individual DAT changes in the CPu after 14 days of treatment with either APD (Supplementary Figure 2b and Fig. 3b) and in the NAcc with HAL treatment (Supplementary Figure 3d). HAL treatment also decreased NET expression in the NAcc (Supplementary Figure 4c). No other effects of HAL treatment on NET or SERT expression were observed in the CPu, NAcc, or PFC compared with veh (Supplementary Figure 4, 5a–f). HAL and its metabolites directly interact with the DAT and other monoamine transporters [35]. Therefore, we further investigated the effect of APDs on DAT regulation. We estimated the putative coupling of striatal TH and DAT expression, which are coregulated [36]. We found no linear relationship between the TH and DAT expression levels in the control animals (Fig. 3d and Supplementary Figure 6a) or short-term HAL (Supplementary Figure 6b) or OLA treatments (Supplementary Figure 6c). However, long-term treatment with HAL (6–14 days) or OLA (21 days) progressively linearized the TH-DAT relationship, and higher TH levels were associated with increased DAT levels (Fig. 3a–c). This result suggests that APD treatment failure occurs in conjunction with the simultaneous increase in capacity for dopamine synthesis and uptake.

Next, we assessed whether changes in DAT levels occurred due to direct effects of APDs on mRNA expression. We measured DAT mRNA expression using polymerase chain reaction in the substantia nigra pars compacta (SNc) and ventral tegmental area (VTA) at different HAL treatment times. We found that HAL suppressed DAT mRNA expression in the SNc and VTA after 2 days (Fig. 3e, f) compared with the control treatment. However, the inhibitory effect of HAL in the SNc gradually subsided during ongoing treatment (Fig. 3e). The suppression of DAT expression by HAL persisted until day 6 in the VTA (Fig. 3f). By day 14, DAT mRNA expression showed a modest trend toward regaining normal levels (Fig. 3f). These data suggest that the efficacy of HAL during short-term treatment is associated with a robust suppression of DAT mRNA expression in the SNc and VTA. In contrast, during APD failure, DAT mRNA expression was restored in the nigrostriatal system but not in the mesolimbic dopamine system. This effect suggests a dissociated mechanism in the brain, with dopaminergic projection systems serving to promote APD treatment failure, and the nigrostriatal system exhibiting the most striking changes.

To further investigate the role of the DAT in APD treatment failure, we measured the DAT density in vivo by microPET imaging using [^18^F]FP-CMT in rats at baseline and after 14 d of HAL treatment. To verify treatment failure at that time, we also measured the TP-induced locomotor activation. We detected increased DAT availability (binding potential; BP_ND_) in striatum at 14 days of HAL treatment (Fig. 3g, h) when treatment failure became evident as a disinhibition of TP-induced locomotion (Fig. 3j and supplementary Figure 7). DAT BP_ND_ correlated with TP-induced locomotion (Fig. 3i), which demonstrates an association of APD efficacy and failure at the behavioral level with a dysregulation of dopamine clearance.

### Electrophysiological activity of dopaminergic neurons during antipsychotic treatment efficacy and failure

**Fig. 4:**
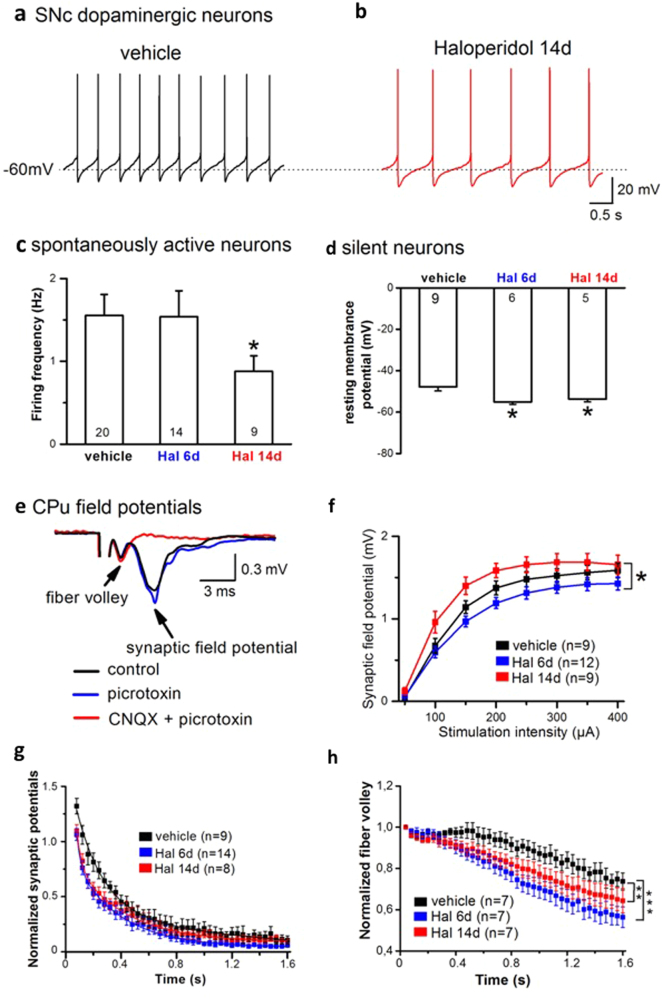
Electrophysiological activity of dopaminergic neurons during antipsychotic treatment failure. Chronic haloperidol (HAL) inhibits substantia nigra pars compacta (SNc) dopaminergic neurons (**a–d**) and changes synaptic transmission in the caudate-putamen (CPu) (**e–h**). **a–b** Representative voltage traces recorded from mouse brain slices show the regular discharges of spontaneously active dopaminergic neurons from a vehicle (veh)-treated (**a**) and a 14-day HAL-treated (**b**), HAL 14d) mouse, respectively. **c**, **d** Histograms summarize HAL-induced reduction in firing rate (HAL 6d *P* = 0.97, HAL 14d *P* = 0.040; vs. veh) and shift of resting membrane potentials in SNc dopaminergic neurons (HAL 6d *P* = 0.047, HAL 14d *P* = 0.020; vs. veh). **e** Superimposed traces recorded from a vehicle-treated mouse slice illustrate the biphasic field potentials (at stimulation intensity of 100 µA) in the CPu, with an early axonal component (fiber volley) and a late synaptic component. Stimulation artifact is truncated. The GABA_A_ receptor antagonist picrotoxin (100 µM) and AMPA receptor antagonist CNQX (20 µM) were used to determine nature of synaptic field potentials. **f** Input–output curves of synaptic field potentials were plotted to show the time-dependent effect of HAL on CPu synaptic transmission (treatment: *P* = 0.012; time: *P* < 0.0001; interaction: *P* = 0.112; two-way ANOVA). **g** The use-dependent decline of CPu synaptic responses during 40 stimuli at 25 Hz was facilitated by HAL treatment. Decay time constant in vehicle group (*n* = 9, 0.31 ± 0.05 s) was significantly longer than in HAL 6d (*n* = 14, 0.18 ± 0.02 s, *P* = 0.030) and HAL 14d (*n* = 9, 0.19 ± 0.02 s, *P* = 0.048). Synaptic responses were normalized to their amplitudes before the train. **h** Chronic HAL reduced the isolated axonal activity during train stimuli. In this set of experiments, fiber volleys were recorded in low calcium solution (0.2 mM) with antagonists for glutamatergic and GABAergic receptors. Amplitudes of fiber volleys were normalized to their amplitudes before train. All data are means ± S.E.M. (*P < 0.05). Sample numbers in each group were indicated in the columns or as insets

Extracellular dopamine levels in nigrostriatal and mesolimbic areas also depend on tonic and phasic dopamine neuron activity [37, 38]. Tonic activity is characterized by a spontaneous slow and irregular firing of dopamine neurons, and phasic activity is mediated by burst firing [39, 40]. We measured the spontaneous firing, resistance, and capacitance of dopaminergic neurons from the SNc in brain slice preparations of mice after 6 or 14 days of HAL treatment (i.e., efficacy and failure conditions). Membrane input resistance and membrane capacitance of SNc dopamine neurons were unchanged after either HAL treatment duration (data not shown). Notably, treatment failure after 14 days of HAL treatment coincided with a significant reduction in the firing rate of spontaneously active neurons compared with the vehicle-treated group, but 6 days of HAL treatment did not alter the firing frequency (Fig. 4c). The slowed tonic firing after 14 days of HAL treatment may at least partially account for the reduced extracellular dopamine levels observed in the microdialysis experiments (Fig. 2e). Both drug regimens also affected silent dopamine neurons and shifted their resting membrane potentials to significantly more negative values (Fig. 4d). The HAL-associated hyperpolarization would impede the transition to tonic firing and impair the phasic mode of firing because only spontaneously active dopamine neurons can burst fire [39]. To examine the impact of the HAL-dampened firing frequency of SNc dopamine neurons on the postsynaptic side, we monitored the neuronal population responses in CPu, the main projection target. Local electrical stimulation in the CPu evoked responses that consisted of a biphasic field potential, with an early axonal component (fibre volley) and a late synaptic component. The latter component predominantly arose from excitatory postsynaptic currents, as indicated by their suppression by the AMPA receptor antagonist, CNQX (Fig. 4e). The effect of HAL on the input–output relationship of synaptic field responses (stimulation intensity 50–400 µA) revealed a striking dependence on treatment duration, with a reduction after 6 days and a potentiation after 14 days of HAL treatment (Fig. 4f). This shift may reflect a reversal of drug action from therapeutic to non-effective. To determine the drug’s effect on synaptic transmission during the repetitive activation of presynaptic fibres, we delivered a train of 40 stimuli at 25 Hz. This stimulation protocol produced an initial enhancement of synaptic field potentials followed by a rapid decline, which was significantly accelerated in slices from 6 days HAL and 14 days HAL mice (Fig. 4g). Consistent with our previous findings, which indicated that the release of an intravesicular pool of accumulated APDs dampens synaptic transmission by blocking presynaptic Na^+^ channels [17], we found that in the same stimulation protocol, the fibre volley amplitudes declined during prolonged HAL administration (Fig. 4h).

### Dopamine release mechanisms during antipsychotic treatment efficacy and failure

**Fig. 5:**
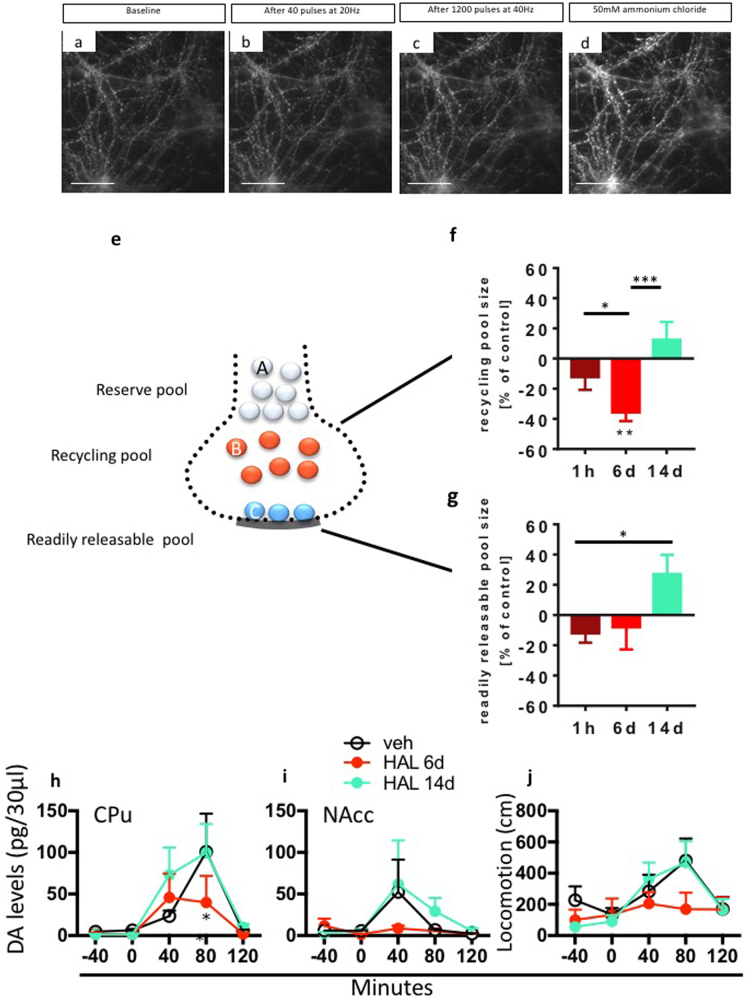
Dopamine release mechanisms during antipsychotic treatment efficacy and failure. **a–d** Exemplary raw recording of neurons during vesicle pool size measurement. Cells were transfected with synaptopHluorin and treated with 80 nM haloperidol (HAL) prior to the experiment. Cells were perfused with imaging buffer containing 80 nM HAL during the entire recording as well; **a** Raw image showing baseline fluorescence; **b** Raw image showing fluorescence after electrical stimulation with 40 pulses at 20 Hz, which corresponds to the readily releasable pool vesicles being fluorescent; **c** Raw image showing fluorescence after further electrical stimulation with 1200 pulses at 40 Hz, which corresponds to the recycling pool vesicles being fluorescent additionally; **d** Raw image showing fluorescence during perfusion with imaging buffer containing additional 50 mM ammonium chloride, which corresponds to all synaptic vesicles being fluorescent; Scale bar = 25 µm. **e** Schematic drawing of the different synaptic vesicle pools in a synaptic bouton: resting pool, recycling pool and readily releasable pool docked to the active zone. **f** Effect of HAL in vesicle recycling pool size in hippocampal cells after 1 h (*P* = 0.0132, *n* = 9 recordings) or 6 (*P* = 0.0052, *n* = 14 recordings) and 14 days (*P* = 0.0009, *n* = 21 recordings) treatment compared with vehicle (DMSO, control group). **g** Effect of HAL in vesicle readily releasable pool size in hippocampal cells compared with controls (*P* = 0.0136, *n* = 21 recordings). **h–i** HAL (0.5 mg/kg/d) treatment for 6, but not for 14 days, reduced potassium (K^+^)-induced (100 mM, infused via reversed dialysis) dopamine release in the caudate-putamen (CPu) (*P* < 0.0001, main effect) and in the nucleus accumbens (NAcc) (*P* < 0.05) compared with baseline *n* = 4–6/group. **l** HAL (0.5 mg/kg/d) treatment for 6, but not for 14 days reduced K^+^-induced (100 mM) hyperlocomotion compared to baseline (*P* < 0.05, main effect). *N* = 4–13/group. All data are means ± S.E.M *P < 0.05, **P < 0.01, ***P < 0.001. Statistical significance represents post hoc comparison when not specified

Active and spontaneous dopamine release occurs via vesicular exocytosis at active zones associated with Ca^2+^ channels [41–44], and APDs target this mechanism [17, 45]. We previously observed that APDs accumulate in the synaptic vesicles of cultured hippocampal neurons via an acidic trapping mechanism and inhibit Na^+^ channels upon release. This inhibition leads to the feedback inhibition of Ca^2+^ influx and reduces neurotransmitter release [17]. We investigated whether longer APD treatment regimens that mimic APD efficacy and failure would affect dopamine exocytosis. We transfected cultures of hippocampal neurons, which should exhibit similar vesicular release mechanisms to dopamine neurons [46], from juvenile rats with synapto-pHIuorin (spH) and recorded synaptic vesicle exocytosis using real-time fluorescence microscopy after 1 h, 6 days or 14 days of treatment with HAL or vehicle control in culture [47]. We measured the sizes of two distinct vesicle pools, the readily releasable pool (RRP) and the recycling pool (RP) [48]. The RRP defines the neurotransmitter pool that is immediately available for release, and RP is related to the replenishment of the RRP during neuronal activity. We found that HAL suppressed the RRP within 1 h of treatment, potentiated suppression after 6 days of HAL treatment, and enhancement of RRP after 14 days of HAL treatment (Fig. 5g). RP was significantly reduced after 6 days of HAL treatment but was enlarged after 14 days of HAL treatment (Fig. 5f). RRP and RP reflect synaptic efficacy under physiological conditions. Therefore, our data suggest that the physiological mechanisms of exocytosis-mediated neurotransmitter release are modulated in opposing directions after HAL treatment during APD efficacy and failure. The present data extend our previous observation that APDs inhibited vesicle release during efficacious treatment, but this effect was reversed with prolonged HAL treatment. Therefore, treatment failure is associated with enhanced dopamine exocytosis.

The exocytosis inhibition in cultured neurons acutely treated with HAL may be attributed to the ability of HAL to inhibit Na^+,^ rather than Ca^2+^, channels because K^+^ treatment induced exocytosis in the presence of APDs [17]. To evaluate whether the same mechanism occurs both in vivo and after treatment with clinically equivalent doses, we measured the extracellular dopamine levels in the CPu and in NAcc in freely behaving rats. Local dopamine release was stimulated using reverse dialysis with 100 mM K^+^ [17] in animals treated for 6 or 14 days with HAL or veh. The 6d HAL treatment blocked the K^+^-induced dopamine release in the CPu (Fig. 5h) and NAcc (Fig. 5i) and prevented K^+^-induced hyperlocomotion (Fig. 5j). These effects were reversed after 14 days of HAL treatment (Fig. 5h–l), consistent with the effects of APDs on vesicle pool parameters because HAL treatment reduced stimulated dopamine release in the brain during the treatment efficacy phase but not during treatment failure.

### Reversal of antipsychotic treatment failure

**Fig. 6:**
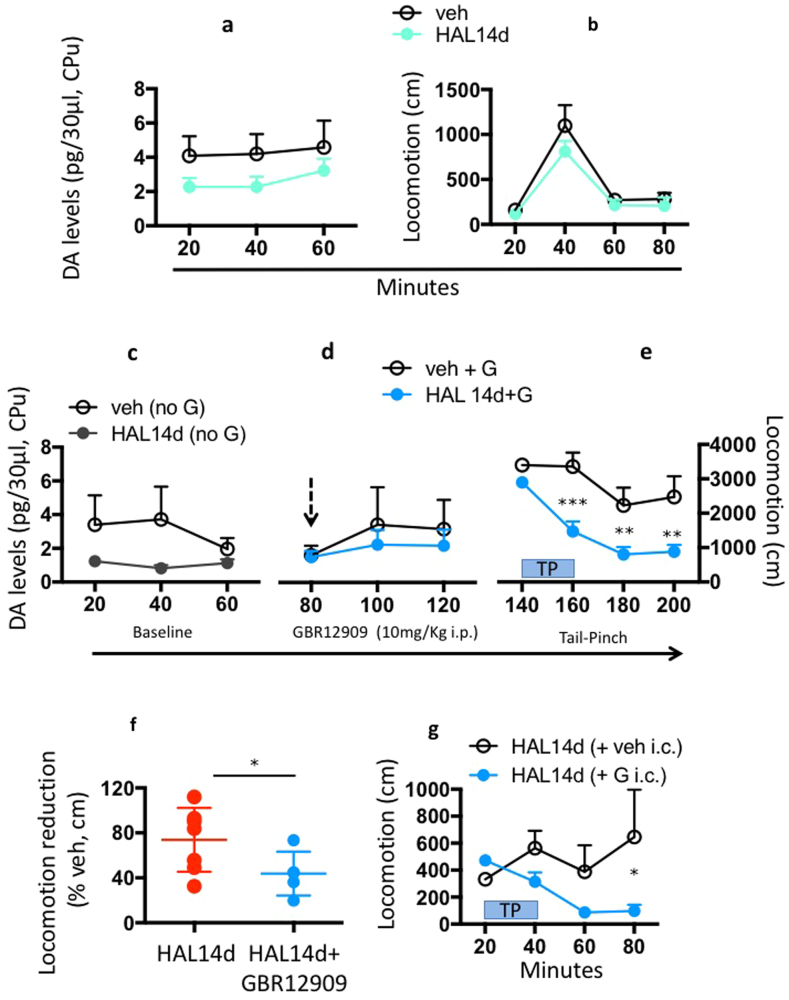
Reversal of antipsychotic treatment failure. **a** Haloperidol (HAL) treatment for 14d decreased the extracellular dopamine levels in the caudate-putamen (CPu) (*P* < 0.05, main effect) and **b** is unable to block tail pinch (TP) induced hyperlocomotion (*P* < 0.0001, main effect) compared to vehicle (veh) treated rats. *N* = 7/group. Data are means ± S.E.M. **c** Similar to the previous experiment, HAL decreased the extracellular dopamine levels in the CPu compared with veh (*P* < 0.05, main effect). **d** The systemic injection of GBR12909 (10 mg/kg, i.p.) replenished the extracellular dopamine to control group levels, and **e** reversed HAL failure, which was now sufficient to block TP induced hyperlocomotion compared to veh (*P* < 0.0001, main effect). *N* = 5/group. Data are means ± S.E.M. **f** The co-administration of GBR12909 (10 mg/kg, i.p.). *N* = 5–7/group. Data are means ± SD. **g** The intraCPu injection of the DAT blocker GBR12909 decreased the TP-induced hyperlocomotion in animals treated 14 days with HAL compared with control animals (*P* < 0.05, main effect), thus mimicking the effect observed under systemic treatment of GBR12909. *N* = 4/group. Data are means ± S.E.M. *P < 0.05, **P < 0.01, ***P < 0.001. Statistical significance represents post hoc comparison when not specified

APD treatment failure and the generally variable responses to APD treatment are major problems in schizophrenia pharmacotherapy. Having identified the mechanisms that may mediate unfavourable outcomes, we next derived a potential intervention to reverse APD treatment failure and reinstate APD efficacy in behavioral and neurophysiological terms. The present data suggest that if reduced tonic dopamine transmission in the striatum is relevant to the loss of APD efficacy, then a moderate stimulation of tonic dopamine release may restore APD efficacy. We tested this hypothesis in microdialysis studies in freely moving rats. One study measured the extracellular dopamine levels in 14 days HAL-treated and control animals and their locomotor response to TP. We also performed an identical study with an additional treatment of both animal groups with the DAT blocker GBR12909 (10 mg/kg, i.p.) after measuring dopamine at baseline and before recording the TP-induced hyperlocomotion. As observed previously, HAL reduced the extracellular dopamine levels after 14 days of treatment compared with the control treatment (Fig. 6a) in association with the failure of HAL to inhibit the TP-induced hyperlocomotion (Fig. 6b). We replicated the finding of reduced extracellular dopamine during prolonged HAL treatment (Fig. 6c). The co-administration of the DAT blocker moderately increased extracellular dopamine levels in 14 days HAL-treated animals but not in the control group, which reduced the group differences in dopamine levels (Fig. 6d). The combination of the acute DAT blocker and 14 days HAL treatments resulted in a net inhibition of TP-induced hyperlocomotion (Fig. 6e). Therefore, GBR12909 rescued ~40% of the inhibitory effects of HAL (Fig. 6f). This behavioral effect was not confounded by stereotypies, which may occur when GBR12909 or other psychostimulants are administered after chronic APD regimens [49]. The animals in our conditions exhibited a “fluid” execution of locomotor activity that was not compromised by stereotypes, which would otherwise rigidly dominate the overall behavioral performance. The dose of GBR12909 only modestly increased extracellular dopamine levels (Fig. 6d). These results clearly suggest that a slight potentiation of tonic dopamine levels rescued APD treatment outcomes, which supports the hypothesis that DAT blockers may be used clinically as an adjunct treatment to reverse APD treatment failure.

We also investigated whether the mechanism of this protective effect was mediated in the CPu. We administered GBR12909 (G, 20 µg/µl/side) or vehicle directly into the CPu of rats with 14 days of HAL treatment. Intracerebral vehicle infusion did not alter the usual loss of locomotor inhibition in HAL-treated rats (Fig. 6g), which is indicative of treatment failure. However, the infusion of GBR12909 to the CPu (intra-cuadate, i.c.) inhibited the TP-induced locomotion in 14 days HAL-treated animals (Fig. 6g), which mimicked the behavioral response that is normally observed during HAL treatment efficacy. These data strongly suggest that the dopaminergic innervation of the CPu is a critical site for a pharmacological reversal of APD treatment failure.

## Discussion

The present findings elucidate crucial mechanisms of APD therapeutic action and failure and provide a new strategy for failure reversal. First, our model reproduced APD action and failure using clinically relevant APD dosing and treatment regimens. Second, the mechanism underlying APD efficacy was primarily characterized by preserved or increased extracellular dopamine levels in the CPu in association with reduced DAT mRNA expression and relatively preserved expression of TH and of membrane DAT protein. Third, the physiological pattern of APD efficacy was characterized by preserved tonic firing of SNc dopamine neurons, decreased post-synaptic responses (field potential) and suppression of exocytosis (recycling pool size). The physiological changes underlying APDs efficacy were substantially reversed during APD failure. Specifically, we found that (1) baseline extracellular dopamine levels in the CPu were suppressed, (2) the initial inhibition converted into an upregulation of both DAT mRNA and membrane protein, and (3) neurotransmitter synthesis and release mechanisms were potentiated. However, we also found that at a time interval when we recorded APD failure, i.e., after 14 days of treatment, D_2_ receptor occupancy to [^18^F]fallypride PET was in a “therapeutic range,” as measured in an independent group of animals. These results suggest that mechanisms beyond D_2_ receptor occupancy and depolarization blockade are involved in APD treatment efficacy and failure. Our data suggest a specific role of the DAT in the mediation of APD treatment efficacy and APD failure. Therefore, dysregulated dopamine uptake, which follows the initial suppression of DAT expression, leads to decreased extracellular dopamine levels. Informed by these observations, we reinstated the behavioral parameters of APD efficacy using pharmacological blockade of the DAT. Therefore, human patients experiencing relapse upon chronic treatment with APDs may experience restored therapeutic efficacy when APD treatment is combined with DAT blockers, such as Vanoxerine (GBR12909) [50], which have the advantage of evoking only a moderate increase in extracellular dopamine, despite acting at a higher binding affinity than cocaine, thus perhaps lowering the risk of abuse [51, 52].

Conflicting evidence exists for the role of DAT in schizophrenia [53, 54], with most of the studies reporting no change [55] or a decrease in DAT density in terminal areas of dopaminergic neurons, especially in patients treated with APDs [55–58]. These discrepancies could be attributed to independent but overlapping factors. For example, the choice of testing drug-naïve patients can itself be a source of variability since genetic heterogeneity drives DAT expression [59]. Based on our results, DAT density alterations can be driven solely by APD treatment (Fig. 3h, j and supplementary Figures 2b and 3b). The age of patients is an additional factor that might introduce variability and limit the detection of DAT density changes in human studies. PET imaging studies have shown significant differences in DAT density between <40 vs. >40 years old healthy individuals [60]. Most likely, DAT density will not change at the same rate on a particular time point in all individuals. Rather, it will fall below some threshold at a definite age, e.g., <40 years, for each individual. Therefore, a considerable variability in DAT availability might even exist within the same age group.

Furthermore, it has been suggested that brain aging as distinct from chronological age of the organism can alter DAT density via deterioration of white matter integrity and cardiovascular risk factors accounted for the underlying shared mechanism [61]. Notably, white matter integrity is an important moderator of the initial APD response at the start of the treatment [62]. Continued APD treatment for 12 weeks can cause subtle losses of white matter integrity in first-episode psychosis [63].

Additional factors that may account for the variability in detecting DAT density changes in schizophrenia are ascribable to the dynamic of the DAT expression. DAT levels adapt in line with dopamine transmission, decreasing or increasing depending on whether dopamine release is low or high [64]. During acute psychosis, when dopamine levels are expected to be high, DAT levels might be higher than after the psychotic episodes (i.e., recovery). Our findings are in line with those studies reporting on the dynamicity underlying DAT density changes. We demonstrated a considerable suppression of DAT mRNA expression during the initial stages of HAL treatment (after 2 days), which gradually reversed within 2 weeks in the SNc, but remained substantially suppressed in the VTA during the same time period. These changes in DAT mRNA expression reflected corresponding changes in DAT protein expression at the plasma membrane. Furthermore, the HAL efficacy in behavioral tests clearly decreased after the first week of treatment when most of the DAT adaptations had already taken place. This plasticity would have been missed if measurements had been performed only on day 6 or later, i.e., when antipsychotic efficacy was already abolished. Besides indicating critical time points when putative DAT density changes driven by the APD treatment may be detected, our data strongly suggest that the early inhibition followed by a normalization or, in some cases, an upregulation of DAT expression in striatal tissue constitutes a fundamental component of HAL efficacy and failure, respectively. Accordingly, a previous rat microPET study using [^123^I]-FP-CIT has shown reduced DAT binding only minutes after acute HAL injection [65]. Based on our data, the HAL-driven reduction in DAT binding may occur either directly through reduction of DAT density in the plasma membrane or via suppressed DAT mRNA expression at very early treatment stages. Alternatively, availability changes may reflect radioligand displacement by increased dopamine levels due to the inhibition of DAT expression. It is also be possible that sustained brain concentrations of HAL resulting from the treatment with osmotic pumps may block the DAT transporter directly, thus causing an increase in dopamine levels. Studies using neuronal cell culture have shown the likelihood of this possibility [35]. Additionally, the increased dopaminergic tone, driven by the initial HAL treatment, can downregulate DAT membrane expression via the stimulation of the D_2_ autoreceptors [66].

The present data support pharmacogenomics studies reporting associations between *DAT* gene variations and clozapine efficacy in treatment resistant patients and in cognitive dysfunctions [67, 68]. Thus, targeting DAT may be a paramount goal of APDs to overcome treatment-resistance in schizophrenia. A second observation supported by our data relates to our recent suggestions about the actual mechanisms of APD action [5]. We argue that the rise and fall of antipsychotic efficacy is driven by dynamic interactions of the endogenous dopamine and presynaptic D_2_ receptors. Specifically, increased tonic synaptic levels of dopamine in the striatum following the initial treatment with APDs would determine the stimulation of a dopamine D_2_ receptor reserve, which is defined as the difference between the total number of available D_2_ receptors (100%) and the proportion of those D_2_ receptors bound by an APD at a dose within the therapeutic window (60–80% blockade of central D_2_ receptors). The receptor reserve primarily includes presynaptic dopamine receptors, which tonically inhibit dopamine synthesis and release, and may mediate antipsychotic effects [5]. Present results showing an early suppression of mRNA DAT expression (Fig. 3e, f) along with an inhibition of vesicular release during antipsychotic efficacy (Fig. 5f, g) substantiate these interpretations.

By contrast, decreased tonic synaptic availability of dopamine, occurring during chronic APD treatment, is associated with a significant loss of APD efficacy. These effects may reflect a reduced stimulation of the D_2_ receptor reserve triggered by the initial treatment [5]. Notably, dopamine release in the striatum is closely related to the firing of dopaminergic neurons in the midbrain, which was decreased in this study (Fig. 4c). Furthermore, DAT was upregulated during APD failure (Fig. 3e, h), thus, possibly increasing clearance of extracellular dopamine and contributing to a reduction of synaptic dopamine levels.

Reduced stimulation of D_2_ autoreceptors would predict that presynaptic neurons will be more sensitive to phasic dopamine release in response to stimulation. Consistent with this proposal we found increased TH expression (Supplementary Figure 1a, d), a potentiation of vesicular release (Fig. 5f, g) and a larger dopamine release in response to TP in those animals with lower basal dopamine levels (Fig. 2h, i).

Furthermore, local modulatory neurotransmission in the striatum affects release via direct action on axon terminals. Glutamatergic and cholinergic activity is sufficient to trigger striatal dopamine release independently of somatic firing [69]. Therefore, the decrease in synaptic dopamine driven by APDs in the striatum may make dopaminergic terminals more vulnerable to the excitatory effect of local glutamatergic transmission, which may result in increased field potentials (Fig. 4f). Also, synaptic neuroplasticity and related changes in multiple protein expression may pose viable interpretations of the role of APDs in structural brain changes observed in humans and in animals [70].

Our model of APDs efficacy and failure outlines quite a different perspective compared to traditional interpretations of antipsychotic mechanisms. The common view is that an “optimal” level of post-synaptic dopamine D_2_ receptor blockade with antipsychotics attenuates dopaminergic transmission at post-synaptic neurons [4, 71], which ameliorates the positive symptoms of schizophrenia. However, studies show that chronic APD treatment triggers adaptations of post-synaptic dopamine D_2_ receptors, which undergo upregulation [28, 29, 31] and increased sensitivity for local dopaminergic signalling. This circumstance, known as dopamine supersensitivity [25, 72], is the hypothesized mechanism of antipsychotic treatment failure [25, 27, 72].

Yet, changes in D_2_ receptor density are not consistently observed in patients previously exposed to antipsychotics [73]. APD treatment failure also occurs independently of any changes in D_2_ high affinity state receptor density in animal models [27]. In conclusion, whereas reducing the synaptic levels of dopamine has been the primary target of antipsychotics to reduce psychoses, our data suggest an apparent paradox that reduced dopamine signalling at presynaptic D_2_ receptors is the actual cause of antipsychotic failure. Therefore, reverting dopamine levels in the dopaminergic synapse should reinstate APD efficacy. Here, we demonstrated this to be the case by blocking the clearance of synaptic dopamine using the DAT blocker GBR12909. This pharmacological intervention may be tested clinically as an augmentation strategy, alternative to increasing APD dosing or switching drugs, and possible abuse potential derived from this combination of treatments may be managed. Genetic variability in DAT expression as well as the age of patients at the start of treatment will affect APD response even if augmentation with a DAT blocker is used, simply because not all individuals will have a sufficient amount of DAT expression to mediate the necessary DAT blocker efficacy. Thus, an intriguing therapeutic strategy may be proposed based on viral expression of DAT in key brain areas to overcome treatment-resistance and equalize the therapeutic antipsychotic response across patients. Our study delineates mechanisms of APD efficacy and failure. It suggests a new strategy for personalized therapeutic approaches to overcome pathways to relapse, including APD tolerance, dopamine super-sensitivity psychosis, and treatment resistance.

### Limitations of the study

D_2_ receptors exhibit high and low affinity states for dopamine agonists (D_2_^High^, D_2_^Low^) [74], which are not distinguished using current antagonist ligands [75]. Therefore, an increased prevalence of D_2_^High^ activated with APD treatment may lead to the dopamine supersensitivity underlying the antipsychotic loss of efficacy, despite an unaltered absolute total density of D_2_ receptors and net receptor occupancy. A previous study found that APD treatment failure was associated with a greater prevalence of D_2_^High^ receptor, but this effect occurred only with high HAL doses [27]. Therefore, the role of the unbalanced D_2_ affinity state in mediating APD efficacy and failure in animal models remains to be determined.

We report herein that the D_2_ receptor occupancy to microPET was in the clinical therapeutic range with HAL treatment, even though the drug was no longer effective in independent behavioral testing. Thus, we did not explicitly replicate HAL treatment failure in these animals. Our aim was to verify the D_2_ receptor occupancy range during 14 days HAL treatment. The use of behavioral or pharmacological manipulations may have been confounding factors in our study. Such interventions can stimulate striatal dopamine release, thus perturbing D_2_ receptors availability in the HAL occupancy study after 14 days treatment, as shown in previous human PET studies [76].

Dopamine levels decreased after 6 days of HAL treatment, despite continued drug efficacy at that time point in multiple behavioral tests (Fig. 1). However, a decrease in dopamine levels occurred relative to the control group, but not compared with 14 d of HAL, which further lowered dopamine levels. We attribute this effect to possible different temporal kinetics that underlie changes in neurotransmitter levels and behavior. Therefore, reversal of antipsychotic inhibition in behavioral tests may occur with a different time-course than the reversal of extracellular dopamine. Particularly, the dopamine levels may decrease faster than the indices of behavioral APD efficacy. Furthermore, while the use of psychopathological naïve rodents may appear to limit the translational power of the present study, it should be noted that in absence of a clear understanding of the neurobiology of schizophrenia symptoms, which undermines face validity in experimental modelling, our pharmacological model yields evidence for neuroadaptations induced solely by the administration of APDs. These adaptations are very dynamic and time-locked with the onset and offset of antipsychotic efficacy. Nevertheless, the results of the present work may benefit from replications in psychopathological animal models.

## Electronic supplementary material

S7

S2

S3

S4

S5

S6

Figure S1

Supplemental Material
