# Supplementary figures and images for: A dopaminergic mechanism of antipsychotic drug efficacy, failure, and failure reversal: the role of the dopamine transporter
Source: Mol Psychiatry. 2018 Jul 23;25(9):2101–18. doi: 10.1038/s41380-018-0114-5 (PMC7473845; doi:10.1038/s41380-018-0114-5)

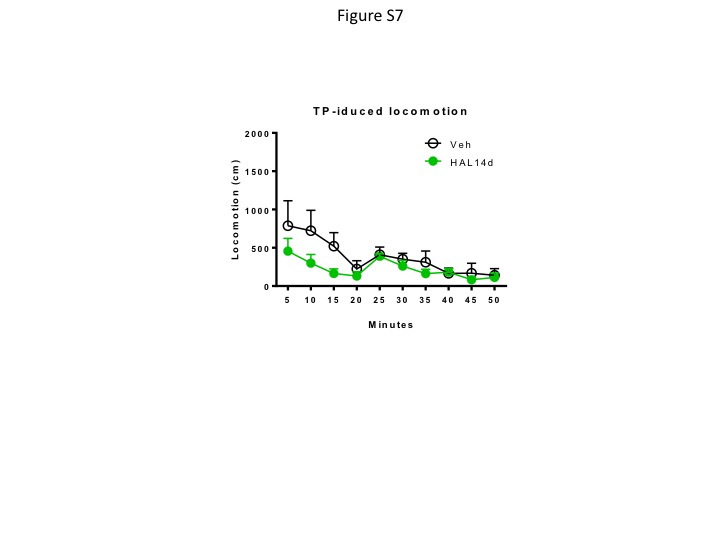

Supplement: Supplementary file 1 — S7 [file 41380_2018_114_MOESM1_ESM.jpg]

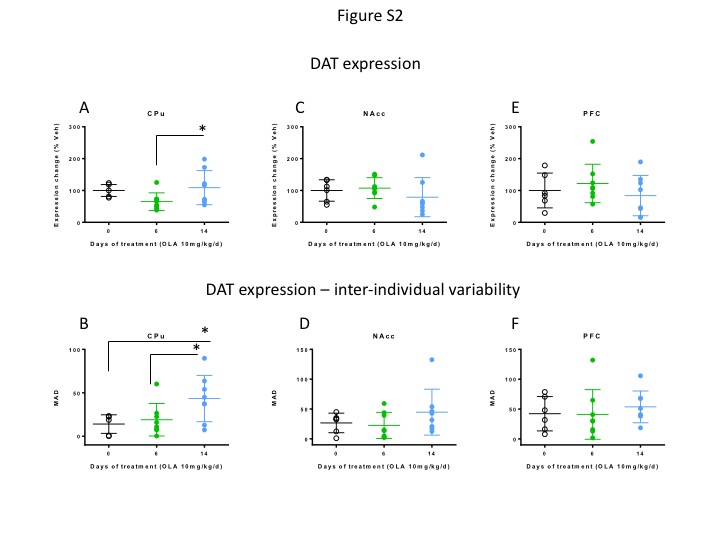

Supplement: Supplementary file 2 — S2 [file 41380_2018_114_MOESM2_ESM.jpg]

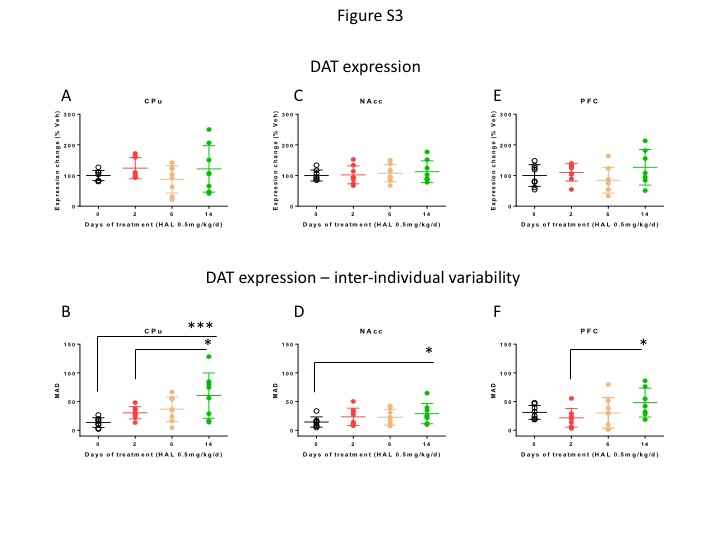

Supplement: Supplementary file 3 — S3 [file 41380_2018_114_MOESM3_ESM.jpg]

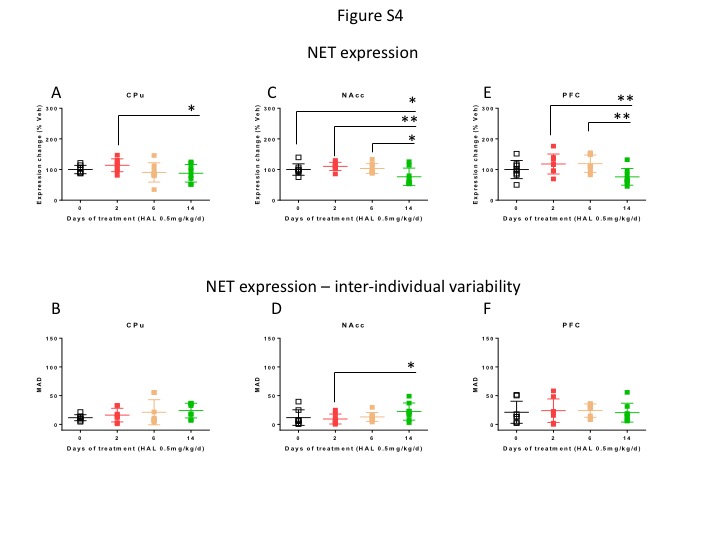

Supplement: Supplementary file 4 — S4 [file 41380_2018_114_MOESM4_ESM.jpg]

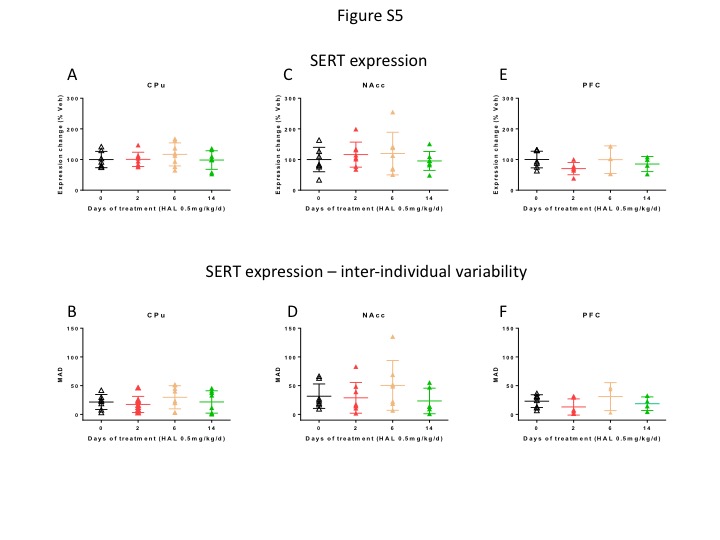

Supplement: Supplementary file 5 — S5 [file 41380_2018_114_MOESM5_ESM.jpg]

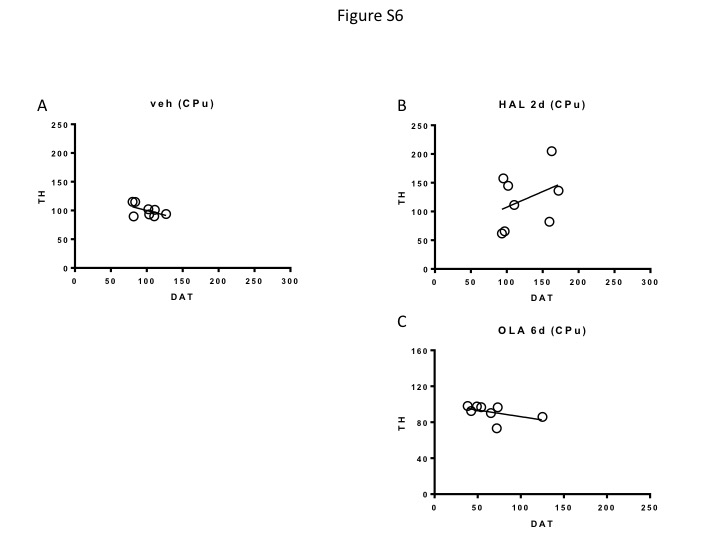

Supplement: Supplementary file 6 — S6 [file 41380_2018_114_MOESM6_ESM.jpg]

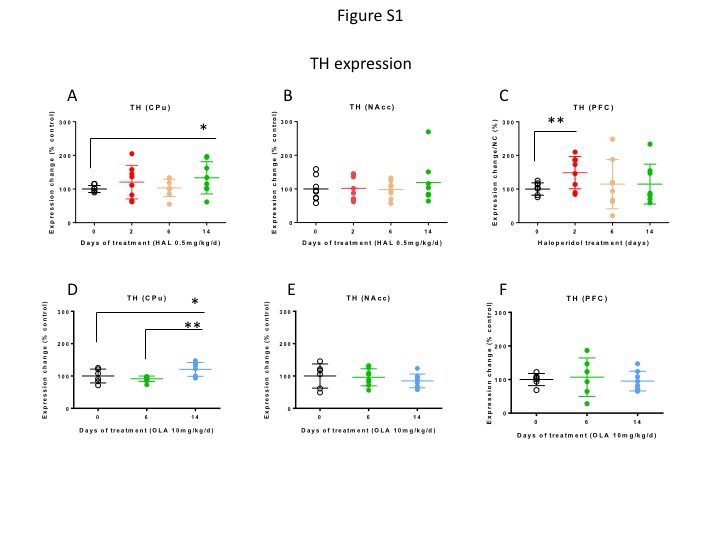

Supplement: Supplementary file 7 — Figure S1 [file 41380_2018_114_MOESM7_ESM.jpg]
