# Supplementary material for: A dopaminergic mechanism of antipsychotic drug efficacy, failure, and failure reversal: the role of the dopamine transporter
Source: Mol Psychiatry. 2018 Jul 23;25(9):2101–18. doi: 10.1038/s41380-018-0114-5 (PMC7473845; doi:10.1038/s41380-018-0114-5)
Supplement: Supplementary file 8 — Supplemental Material [file 41380_2018_114_MOESM8_ESM.docx]

**Material for online version of the paper**

**Materials and Methods**

**Animals**

Male Sprague-Dawley rats (Charles River Laboratories, Germany) weighing 250–300 g were used for most of the studies. Male C57Bl/6 six-week old mice (Charles River Laboratories, Germany) were used for the electrophysiology study only. Upon delivery, animals were socially housed for at least seven days with food and water available ad libitum, while in temperature- and humidity-controlled facilities (22±2°C; relative humidity 55±10%) under a regular 12 h:12 h light/dark cycle (lights on from 7:00 a.m.). All experiments were conducted in accordance with the Animal Protection Law of the Federal Republic of Germany and the European Communities Council Directive of 24 November 1986 (86/609/EEC), and were approved by local authorities. All efforts were made to minimize the number of animals used and to not cause them discomfort.

**Drugs and treatment methods**

Although it is now clear to be not always the case, ^1^ human molecular imaging studies have often suggested that antipsychotic efficacy without excessive extrapyramidal side effects is achieved when D2 receptor occupancy ranges between 65-75 %. ^2, 3^ Building on these still widely-accepted observations, we administered antipsychotics in a continuous fashion via Alzet 2ML2 and 2ML4 osmotic pumps (Charles River Laboratories, Germany) in rats and Alzet 1004 micro-osmotic pumps (Charles River Laboratories, Germany) in mice. The doses of antipsychotics we have given with osmotic pumps have proven effective in obtaining striatal D2 receptor occupancy in the therapeutic range. ^4-7^ These earlier results find support from the present positron emission tomography (PET) study (described in the dedicated section below). Furthermore, the chronic doses of antipsychotics used here do not cause side effects such as catalepsy, ^5, 6, 8, 9^ which are expected to occur when comparable doses are given as an acute treatment. ^10^

Haloperidol (HAL, 0.5 mg/kg/d, Sigma-Aldrich, Germany) was administered at a 5 µl/h flow rate over periods of 2, 6, and 14 days in rats. Olanzapine (OLA, 10 mg/kg/d, Molekula GmbH, Germany) was administered at a flow rate of 5 µl/h over periods of 2, 6 and 14 days, or at a 2.5 µl/h flow rate over a period of 21 days in rats. In mice, the micro-osmotic pumps delivered HAL (HAL, 0.5 mg/kg/d, Sigma-Aldrich, Germany) at a 0.11 µl/h flow rate over periods of 6 and 14 days.

To attain an optimal solubility and stability from degradation, ^11, 12^ which is an issue for olanzapine, ^13, 14^ antipsychotics were dissolved in sterile water vehicle (VEH) containing 0.2 % ascorbic acid and 10% β*-*cyclodextrin (PH=6). D-amphetamine (AMPH, Sigma-Aldrich, Germany) was dissolved in sterile saline vehicle (0.9% NaCl) and administered to rats via intraperitoneal injection (i.p.) at doses of 1, 2 and 3 mg/kg in 1 ml volume to study the optimal dose for the behavioral studies. A challenge of 100 mM potassium chloride (K^+^; Carl Roth, Germany) dissolved in the Ringer’s dialysis solution (final PH 7.4, Delta select, Germany) was delivered for 80 min to the medial prefrontal cortex (mPFC), caudate-putamen (CPu) and nucleus accumbens (NAcc) via reverse dialysis as previously described. ^15^ GBR 12909 (Sigma-Aldrich, Germany), a highly selective competitive inhibitor of the plasma membrane dopamine transporter (DAT) was dissolved in distilled water by gentle heating and sonication. It was administered via i.p. injection at a dose of 10 mg/kg in 5 ml volume, which has mild stimulatory effects on dopamine release ^16, 17^ or locally into the CPu (left and right sides) at a dose of 20 µg in 1 µl volume per side, which was infused over a period of 1 min; the intracerebral cannulas were left on place for a further minute to allow complete diffusion of the drug into the brain parenchyma. ^18, 19^

**Osmotic pumps implantation**

Osmotic pumps containing VEH, HAL or OLA were implanted under anesthesia with 3.5% isoflurane in oxygen. A 2.0-cm-wide incision was made in each animal’s lower back, and a straight tipped hemostat was used to loosen connective tissue between the scapulae. Preloaded minipumps were wiped with 70% isopropyl alcohol, and positioned between the scapulae, with the flow moderator directed away from the skin incision, which was closed using sterile 9 mm surgical staples. ^8, 9^ An antiseptic aluminum formulation (Aluspray® 210 ML) was used to prevent local infections.

**Guide cannula implantation**

Rats were anaesthetized with a mixture of ketamine (0.9 mg/kg), medetomidine (0.4 mg/kg) and sterile water, with additional novaminsulfon (0.1 mg, subcutaneous) for analgesia. After identifying bregma, ^20^ three guide cannulae (MAB 6.14., Microbiotech, Sweden) were implanted in each rat, targeting mPFC (AP +2.8, ML±0.8, DV -2.4 mm), CPu (AP +0.0, ML ±3.5, DV -4.0 mm; 10° to the midline) and NAcc (AP +1.6, ML ±2.6, DV -5.8 mm, 10° to the midline) for the microdialysis study. ^8, 9^ Two guide cannulae were implanted in each rat targeting left and right CPu (AP +0.0, ML ±3.5, DV -4.0 mm; 10° to the midline) for the microinjection study. ^18, 19^ Animals were allowed up to five days recovery in their home cages before proceeding with the experiments. Guide cannulas were inserted at least four days before or after osmotic minipump implantation, depending on the treatment schedule.

**Behavioural studies and in vivo microdialysis in freely moving rats**

On the test day, rats were transferred to the behavioral testing room and left undisturbed to acclimatize for about 20 min. The time course of antipsychotic efficacy was then assessed through several behavioral tasks, described as follows:

*Amphetamine-induced hyperlocomotion*

Prior to treatment with either VEH or AMPH, the basal locomotion of independent groups of rats were recorded over 20 min in standard open-field boxes (50x50 cm) made of dark acrylic material, by means of a black-and-white video camera. Next, all animals returned to their home cages and received a challenge of VEH or AMPH (2 mg/kg). Beginning five min after the injection, their locomotion was recorded for a total of 40 min, and quantified using the video-tracking software Viewer3 (Biobserve, St. Augustin, Germany). We monitored the ability of VEH (2-21 days), HAL (2 and 14 days) or OLA (2, 6, 14 and 21 days) to inhibit the hyperlocomotion induced by amphetamine.

*Amphetamine induced pre-pulse inhibition disruption*

The baseline acoustic startle reflex (ASR) and pre-pulse inhibition (PPI) of the startle reflex were measured in rats a few days prior to injections with VEH or AMPH (3 mg/kg), using a Startle Response System (TSE Systems GmbH, Bad Homburg, Germany), as recently described. ^21^ Animals were tested in individual soundproof boxes after being placed with a restraining metal cage (27x9x10H cm) that was tightly anchored to a piezo-accelerometer, allowing for the accurate recording of movements of the animal inside the cage. Two loudspeakers positioned at the right and left of the restraining unit delivered the test noises according to protocols that were designed and controlled by a computer. White noise background was played throughout the testing procedure at a constant intensity of 68 dB. Each testing protocol started after 4 min of habituation to the testing environment, during which time the background noise was also played. ASR was first measured in all experimental animals on two independent occasions, to habituate the rats to the testing procedure while collecting baseline startle data. Animals were exposed to ten repetitions of three different startling stimuli (95, 105 and 115 dB white noise, 20 ms), randomly presented at an inter-trial interval (ITI) of 6-12 s. ASR magnitude to each noise presentation was averaged and expressed in grams. PPI was measured with a protocol consisting of 10 initial repetitions of a startling stimulus (118 dB, 20 ms) followed by a randomly distributed combination of 10 of the same startling trials and 10 repetitions of an inhibitory trial, where a prepulse (80 dB, 20 ms) was played 100 ms before the startling stimulus (118 dB, 20 ms). This shortened version of the PPI test was selected after comparing the results obtained with a standard protocol used previously, ^22^ aiming to give a very timely and precise measurement of the effect of the acute administration of AMPH on sensory-motor gating. ASR magnitude to the initial 10 startling trials was calculated, and PPI defined as the percent inhibition of the ASR in the presence of the pre-pulse: 100-[(PPI/ASR)*100]. We monitored the ability of veh, HAL (2 and 14 days) or OLA (2 and 14 days) to inhibit the PPI disruption induced by amphetamine

*Microdialysis and Behaviour*

To allow for a better stabilization of the neurochemistry recording during the experiments, rats received 15-kDa molecular weight cut-off microdialysis probes (MAB 6, Microbiotech) with a permeable membrane length of 2 mm (mPFC and NAc) or 3 mm (CPu) under a brief anesthesia with isoflurane (3%) the night before the experiment and perfusion with the artificial cerebrospinal fluid (aCSF; Delta select, Germany) occurred at the lowest flow rate. ^8, 9^ The next day, the perfusion rate was increased up to 1.5 ml/min 90 min before sampling every 20 min. Experimental recordings followed up the latter stabilization period. Locomotion was monitored throughout the microdialysis experiments with the TruScan system (Coulbourn, USA) according to methods described previously. ^8, 9, 23^

Microdialysis samples were deep frozen (-80 °C) immediately after collection and analyzed using high-performance liquid chromatography with electrochemical detection (HPLC-EC). The column was an ET 125/2, Nucleosil 120-5, C-18 reversed phase column (Macherey & Nagel, Germany) perfused with a mobile phase composed of 75 mM NaH_2_PO_4_, 4 mM KCl, 20 mM EDTA, 1.5 mM SDS, 100 ml/l diethylamine, 12% methanol and 12% acetonitrile adjusted to pH 6.0 using phosphoric acid. The electrochemical detector (Intro, The Netherlands) was set at 500 mV vs. an ISAAC reference electrode (Antec, The Netherlands) operating at 30 °C. The detection limit of the assay was 0.1 pg, with a signal-to-noise ratio of 2:1. Neurochemical data were not corrected for recovery. ^8, 9^

*Olanzapine efficacy: Tail-pinch-induced dopamine release and hyperlocomotion*

After a stabilization period, we collected baseline microdialysis samples from PFC, CPu and NAcc for 60 min. Subsequently, we applied a Mohr clip 2.5 cm to rats’ tail tip (tail pinch, TP; 20 min application) and collected additional samples for 60 min (Figure S1). We analyzed dopamine with HPLC-EC and recorded locomotion throughout the microdialysis experiment. We monitored VEH and OLA efficacy to block TP-induced dopamine release and locomotion after 2, 6 and 21 days of treatment.

*Haloperidol efficacy: Potassium-induced dopamine release and hyperlocomotion*

This microdialysis study was performed according to the procedure described above and previously. ^15^ After a stabilization period, we collected baseline samples from PFC, CPu and NAcc for 80 min. Thereafter, we replaced the regular aCSF with a K^+^-potentiated Ringer’s solution and infused it through the microdialysis probes for 80 min. Next, the perfusion medium was switched back to normal aCSF and samples were collected for an additional 60 min. We analyzed dopamine with HPLC-EC and recorded locomotion throughout the microdialysis experiment. We monitored VEH and HAL efficacy to block K^+^-induced dopamine release and hyperlocomotion after 6 and 14 day treatments.

*Haloperidol efficacy: Effect of systemic GBR12909*

In this study, we performed two microdialysis experiments according to procedures described above. Experiment 1: After a stabilization period, we collected baseline samples from CPu for 60 min. Subsequently, we applied a TP and collected additional samples for 60 min (Figure S2) Experiment 2: After a stabilization period, we collected baseline samples from CPu for 60 min. Thereafter, we injected GBR 12909 intraperitoneally and continued sampling for 60 min. We next applied TP and collected samples for an additional 60 min (Figure S3). We chose to use TP instead of a stronger stimulant (like AMPH) to prevent confounding pharmacological effects of AMPH and GBR12909. As above, we analyzed dopamine with HPLC-EC, recorded locomotion throughout the microdialysis experiments and monitored the efficacy of VEH and HAL to block TP-induced dopamine release and hyperlocomotion after 14 day treatments.

*Microinjections*

This study was performed according to a procedure described previously. ^18, 19^ Rats were acclimated to the infusion procedure for three consecutive days before commencing the experiment. Immediately before starting the experimental session, we removed the stainless-steel stylets from the guide cannulae and replaced them with custom made stainless steel injectors, which extended 1 mm beyond the tip of the guide cannulae targeting brain areas (left and right CPu).

*Haloperidol efficacy: Effect of intrastriatal GBR12909*

On testing day, we placed rats in Truscan locomotion arena for 30 min to allow abituation. Subsequently, either veh or GBR12909 were infused to the cannulae in rats treated with VEH or HAL for 14 days, respectively. Three minutes later we applied TP and recorded locomotion for a total of 60 min.

**Western Blot Study**

*Neuroplasticity induced by Antipsychotics: Determination of Monoamine transporters and Tyrosine hydroxylase*

*Whole-tissue*

Dopamine, serotonin and noradrenaline transporter (DAT, SERT and NET, respectively) protein expression levels were measured by standard western blotting in brain samples from rats treated with chronic veh or HAL (Figure S4). Subsequently we measured the DAT expression levels after treatment with OLA (Figure S5). Groups of rats were killed by decapitation at 2, 6 and 14 days of treatment, and brains were rapidly excised and dissected on ice, according to published methods ^24^ into the region of interest (CPU, NAcc and PFC). Weighed brain specimens were stored at – 80°C until analysis. Samples were homogenized by sonication for 10 secs in 10 volumes (vol/w) of Triton X-100 buffer (10 mM Tris adjusted to pH 7.4, 150mM NaCl, 1mM EDTA, and 1% v/v Triton X-100, supplemented with complete Mini protease inhibitor mix (Riche, Germany). Total protein content was determined in all homogenates using the Bradford reagent (Roti®-Quant, Roth, Karlsruhe, Germany) and colorometric assay. Portions containing 100 µg of total proteins were loaded onto a Tris-Acetate 3-10% polyacrylamide gel (BioRad), electrophoresed for 1.5 h at 100 V, and then transferred onto PVDF membranes at 1 5V for 30 min. DAT, SERT and NET proteins were detected by first incubating the PVDF membranes overnight at 4°C with rabbit anti-rat DAT, SERT and NET polyclonal antibody (Chemicon), diluted 1:500 in TBS-T containing 5% v/v skim milk. After washing the membranes with TBS-T, anti-rabbit horseradish peroxidase-conjugated secondary antibody (1:3000, SantaCruz) was applied for 1 h at room temperature. DAT, SERT and NET immunoreactive bands were finally visualized on the membranes after incubation with Luminata forte (Millipore) for one minute in the dark, using a ChemiDoc Imager System (BioRad). Membranes were then stripped and probed for tyrosine hydroxylase (TH) and GAPDH, as a loading control, using monoclonal antibodies (1:1000, Chemicon). Band intensities were normalized relative to the arbitrary densitometric values obtained for control, untreated, animals, thus expressed as fold/percent change in DAT, SERT, NET and TH expression.

**Polymerase Chain Reaction Study**

*Neuroplasticity induced by antipsychotic drugs: Determination of nigro- and meso- striatal DAT mRNA*

DAT mRNA expression levels were quantified in freshly-dissected portions of striatum according to a standard reverse transcriptase-coupled quantitative real-time PCR (qRT-PCR) procedure using the primer pairs DAT-for-agctaccatgccctatgtgg and DAT-rev-atcagcactccaaacccaac. In brief, total RNA was extracted from tissue using RNA-Pure (PeqLab, Erlangen, Germany) antero-transcribed into cDNA with First-Strand reagent (Invitrogen, Life Technologies, Darmstadt, Germany). qPCR reactions were then performed on an iQ5 thermo-cycler (BioRad, Munich, Germany) using SYBR-Green reagent (BioRad, Munich, Germany) in a final volume of 25 μl. The relative amount of each transcript was calculated using the cycle threshold method, after normalization relative to expression of a “housekeeping” gene, rpl13a (rpl13a-for-GCTGCCGAAGATGGCGGAGG and rpl13a-rev- CACCTTTCGGCCCAGCAGTACC).

**Radiosyntheses**

*Radiosynthesis of [^18^F] fallypride*

The [^18^F]fallypride radiosynthesis was performed as described previously, ^25^ with some modifications. A QMA-cartridge preloaded with [^18^F]fluoride was eluted with a solution of 15 mg Kryptofix^®^ 2.2.2 and 1 M K_2_CO_3_ (15 µL) in 1 mL acetonitrile. The solution was evaporated using a stream of nitrogen at 85 °C and the labeling precursor (S)-2,3-dimethoxy-5-[3-[[(4-methylphenyl)-sulfonyl]oxy]-propyl]-*N*-[[1-(2-propenyl)-2-pyrrolidinyl]methyl]-benzamide (Tosyl-fallypride, 2 mg, ABX, Radeberg, Germany) dissolved in anhydrous acetonitrile (500 µL) was added. After 5 min at 85 °C, [^18^F]fallypride was isolated by semipreparative HPLC (Kromasil C8, 125 × 8 mm, 4 mL/min, acetonitrile (0.1% TFA)/water (0.1% TFA) 25:75 (v/v), t_R_ = 12 min) followed by solid phase extraction (Oasis HLB cartridge, 150 mg, Waters, Eschborn, Germany). The product was eluted with ethanol (1.5 mL), which was evaporated *in vacuo*, followed by formulation in 0.9% saline. Starting from [^18^F]fluoride (500 MBq), this procedure yielded 250 MBq (radioactivity yield of 50%, decay-uncorrected) [^18^F]fallypride in a total synthesis time of 45 min with a molar radioactivity of 90 GBq/µmol.

*Radiosynthesis of [^18^F]FP-CMT*

The radiosynthesis of [^18^F]FP-CMT was performed as described previously, ^26^ giving the title compound in radioactivity yields of 14-19% (decay-uncorrected) in a total synthesis time of 50 min with molar radioactivities of 14-23 GBq/µmol.

**MicroPET brain studies**

*Determination of striatal dopamine D_2/3_ receptor availability*

Rats treated with VEH (n=4) or HAL (n=4) for 14 days were used for these small animal PET studies. Under isoflurane anesthesia (2%), rats were placed in the aperture of the Inveon small animal tomograph (Siemens Healthineers, Erlangen, Germany) in a dorsal supine position. A dynamic emission recording began upon tail vein injection of [^18^F]fallypride (14-19 MBq, 0.3-1.8 nmol per animal) in a volume of 200 μL saline as a slow bolus. Emission recordings consisted of 23 frames (12 × 10 s, 3 × 1 min, 5 × 5 min, 3 × 10 min), ending at 60 min post-injection, followed by a 7-min attenuation scan. Image reconstruction was exactly as described previously, ^26, 27^ and the decay/attenuation-corrected dynamic emission sequences were converted to MINC format (Montreal Neurological Institute). The summation image was resampled to an anatomic atlas of the rat brain using a nine-parameter rigid body manual registration. The resultant transformation was used to resample the dynamic sequence to the standard coordinates, and a template encompassing the entire cerebellum was used to extract a reference tissue time-activity curve (TAC). Parametric maps of the binding potential (*BP_ND_*) were then calculated by the simplified reference tissue method (SRTM). ^28^

*Determination of striatal DAT binding potential and relationship with TP-induced locomotion*

Rats treated with VEH (n=4) or HAL (n=12) for 14 days were used for small animal PET studies with the DAT ligand [^18^F]FP-CMT, first at baseline the day prior to implantation of osmotic minipumps, and again on day 14 (follow-up). Dynamic emission recordings lasting 45 minutes were initiated upon intravenous bolus injection of [^18^F]FP-CMT (4-17 MBq), with experimental conditions, reconstruction and image analysis otherwise as described above for [^18^F]fallypride. Pairs of *BP_ND_* maps were calculated relative to the cerebellum TAC as documented in our characterization of this DAT ligand. ^26, 29^ Additionally, we measured the locomotor activity stimulated by a TP in a subset (N= 9 out of 12) of HAL treated rats the day before the follow-up. The locomotion recording lasted 30 minutes in total. To assess the relationship between DAT density and locomotion response to TP, we compared the TP-induced locomotion and the DAT density at follow-up as the delta of baseline to obtain a coefficient of DAT density variation in the population. Subsequently, we generated two subgroups of animals using the median split of the distribution of DAT density coefficient, which was 0.8 (BP). Values equals or inferior to 0.8 went into the subgroup “efficacy prone” and the rest into the subgroup “failure prone” (Figure 3j).

**Brain Slice Electrophysiology**

VEH or HAL treated C57BL6 mice were anesthetized with sevoflurane and brain slices (250-300 µm thick) containing the midbrain or the dorsal striatum were prepared in ice-cold high-sucrose solution of the following composition (in mM): 75 sucrose, 87 NaCl, 3 KCl, 0.5 CaCl_2_, 7 MgCl_2_, 1.25 NaH_2_PO_4_, 25 NaHCO_3_ and 10 D-glucose. Slices were incubated in warmed sucrose-solution (35 ºC) for 10 min and subsequently stored in modified aCSF containing (in mM) 125 NaCl, 3 KCl, 1 CaCl_2_, 3 MgCl_2_, 1.25 NaH_2_PO_4_, 25 NaHCO_3_ and 10 D-glucose at room temperature for at least 2 h before being used. Individual slices were then transferred to a submerged chamber perfused with normal aCSF (1.3 mM MgCl_2_ and 2.5 mM CaCl_2_) at 32 ºC. All solutions were constantly gassed with 95% O_2_ - 5% CO_2_.

Whole-cell recordings of visualized neurons in the substantia nigra pars compacta (SNc) were recorded with patch pipettes filled with (in mM) 135 K-gluconate, 5 HEPES, 3 MgCl_2_, 5 EGTA, 2 Na_2_ATP, 0.3 Na_3_GTP, 4 NaCl (pH 7.3). In general, dopaminergic neurons of the midbrain are heterogeneous in several respects, including anatomy, physiology, pharmacology and their gene expression profiles ^30^. Here, we collected data from ‘conventional’ dopaminergic neurons, which were identified by the following characteristic electrophysiological and pharmacological fingerprints: low-frequency pacemaker activity, broad action potentials followed by a pronounced after-hyperpolarization, a strong sag component upon hyperpolarization mediated by hyperpolarized-activated cation current, and membrane hyperpolarization to dopamine (30 µM) application. ^31, 32^ Action potentials (APs) of SNc dopaminergic neurons were recorded in current-clamp mode. A pipette filled with modified aCSF was used to record field potentials in dorsal striatum, which were evoked via a concentric bipolar electrode located nearby. Signals were filtered at 6 kHz (for AP) or 1 kHz (for field potentials) and sampled at 20 kHz using a Multiclamp 700B amplifier in conjunction with Digidata 1440A interface and pClamp10 software (Molecular Devices, Sunnyvale, CA).

Data analysis was performed off-line with Clampfit (Molecular Devices). Data are expressed as means ± S.E.M. Statistical comparisons of data were performed using ANOVA or Student's t test as appropriate. Significance was assumed for P < 0.05.

**Neurophotonic**

*N. Vesicle pool size measurement*

To probe synaptic vesicle pool sizes, primary hippocampal neurons were cultured and transfected with synapto-pHIuorin (spH). After treatment with haloperidol (or DMSO vehicle) synaptic vesicle exocytosis was recorded with a fluorescence microscope. Acquired images were processed and the sizes of the synaptic vesicle pools were statistically analyzed. SpH transfection is a commonly-used and well-established method to monitor synaptic vesicle exocytosis ^33-35^. It is known, that spH transfection does not perturb presynaptic function and is therefore a suitable staining method to visualize synaptic transmission. ^36^ For spH transfected neurons, containing the fluorophore in their synaptic vesicle lumen an increase of pH also increases fluorescence, whereas a decrease of pH decreases fluorescence.

Since the fluorescence emission of spH is quenched by re-acidification of recycled synaptic vesicles, the v-ATPase inhibitor concanamycin A, also referred to as folimycin, had to be applied during synaptic vesicle pool size measurements. Synapto-pHIuorin molecules in fused vesicles are exposed to the relatively alkaline synaptic cleft milieu and emit fluorescence. In the presence of folimycin , fluorescence emission is not quenched after vesicle recycling, so that vesicle pool sizes measurements are not distorted by those recycled vesicles.

*N.1. Cell Culture und Transfection*

The preparation steps to obtain hippocampal rat neurons were performed as described in. ^37^ Hippocampi were removed from one-to-three day old Wistar rats (Charles River, USA), after they were sacrificed in accordance with the guidelines of the State of Bavaria.

Hippocampal cells were transferred to ice cold Hanks buffered saline solution (HBSS with phenol red and 0.35 g/l NaHCO_3_) and after several washings steps the cells were digested with trypsin (in mM: 215 Trypsin, 137 NaCl, 5 KCl, 7 Na_2_HPO_4_, 25 HEPES, pH=7.5). The reaction was stopped with fetal calf serum (FCS, Life Technologies, Carlsbad, CA, USA) and after further washing steps, cells were dissociated by treatment with MgSO4 (12 mM diluted in HBSS) and mechanical trituration. Cell suspensions were then centrifuged and the supernatant liquid was discarded. The remaining cell pellet was resuspended in ‘starting medium’ (500 ml MEM, 2.5 g glucose, 0.1 g NaHCO_3_, 0.05 g Transferrin, 50 ml FCS, 5 ml 0.2M *L*-glutamine, 1 ml solution of Insulin) and plated on glass cover slips in 12-well-plates, previously coated with Matrigel^TM^ (a combination of several ECM-proteins, BD Biosciences). On the second day *in vitro* (DIV 2), medium was changed to ‘growing medium’ (starting medium with additional 25 ml FCS, 1.25 ml 0.2M *L*-glutamine, 10 ml B27-supplement, 6 μM cytarabine, 535 μl Pen/Strep(100x)).

On DIV 3, the cells were transfected with synapto-pHIuorin ^38^ by a modified calcium phosphate method as described previously. ^37^ For this purpose, cells were incubated with transfection solution (containing 60 μl DNA, 60 μl CaCl2, 480 μl H^2^O, 2xBBS 600 μl, 10.8 ml NBA) for 30 minutes, allowing the calcium chloride to form precipitates. Afterwards, cells were washed (HBSS) and growing medium was added (1500 μl per well). The cells were cultured in an incubator (36°C, 5% CO2, 95%rH) until measurement on DIV 20 to 30.

Prior to measurement, the cells were incubated with 80 nM haloperidol (Sigma-Aldrich, St. Louis, MO, USA), diluted in dimethyl sulfoxide (DMSO; Carl Roth, Karlsruhe, Germany) or pure DMSO (0.1 % [vol/vol]), for 1 hour, 6 days or 14 days respectively.

*N.2. Live-cell fluorescence imaging*

To ensure healthy cell for experiments, samples were chosen after bright field microscopy inspection, excluding coverslips that showed signs of weak vitality (e.g. no clustered soma or detached, loose axons). Coverslips were placed in perfusion chambers, covered with 500 μl of imaging buffer (normal imaging buffer, also referred to as NT, in mM: 144 NaCl, 2.5 KCl, 10 Glucose, 10 HEPES, 2.5 CaCl_2_ and 2.5 MgCl_2_, pH=7.5).

The fluorescence signal of the spH transfected neuronal cell culture was recorded at room temperature in a set-up consisting of a Nikon TI-Eclipse inverted fluorescence microscope, equipped with a 60x, 1.2 NA water immersion objective. To retain focus during perfusion, a Nikon Perfect Focus System^TM^ was used. The fluorescent probes were excited by a Nikon Intensilight C-HGFI through an excitation filter, passing wavelengths of 455 to 485 nm. The emitted light was recorded by a -90°C water-cooled EM-CCD camera (iXonEM DU-885, Andor, Belfast, Norther Ireland), after passing an emission band-pass filter ranging from 500 to 545 nm. The dichroic long-pass mirror had a cutoff wave length of 495 nm (all from Semrock, Rochester, NY, USA).

A constant perfusion rate with imaging buffer (0.5 ml/min) during imaging was carried out by a piezo-controlled perfusion system (Fast-Step Valve Control Perfusion System SF-77B, Warner Instruments, Hamden, CT, USA). Constant fluid levels were maintained by using a fluid level control (MPCU-3, Lorenz, Katlenburg-Lindau, Germany) and a peristaltic pump. Imaging buffer was supplemented with treatment substances according to experimental requirements.

The synaptic boutons were stimulated by electric field stimulation (51 mA for 1 ms, alternating polarity) delivered through two parallel platinum electrodes, spanning a field of 10 mm. Stimulation (STG 4008, Multichannel Systems) was performed in combination with a stimulus isolator (World Precision Instruments, Sarasota, FL, USA). To improve the signal-to-noise ratio, data binning (2 x 2) was used in the captured image. Recordings were exported into tagged image file format (tif), containing the monochromatic pixel values (resolution 500 x 502 pixels).

*N.3. Experimental protocols*

In vesicle pool size measurements, all used imaging buffers were additionally supplemented with haloperidol (80 nM in DMSO) and folimycin (80 nM). For the control group, the appropriate DMSO concentration without HAL was used. After ensuring stable baseline fluorescence (2 min), exocytosis of synaptic vesicles was electrically stimulated with 40 pulses at 20 Hz, evoking exocytosis of the readily releasable pool. ^39^ After allowing fluorescence levels to stabilize (1 min) another electrical stimulus was delivered. 1200 pulses at 40 Hz caused the entire recycling pool to be released. ^39^ Following another stabilization phase (1 min), cells were perfused with imaging buffer containing additional 50 mM ammonium chloride for 20s. De-acidification due to ammonium chloride unquenches spH in all synaptic vesicles (also in the resting pool) so that the recorded fluorescence emission during this treatment corresponds to the total vesicle pool size. ^40^ Images were acquired with an exposure time of 200 ms at a frame rate of 1 Hz and set in windows of 4 Hz frame rate during stimulation.

**Histological Assessment of Cannulae Placement**

After completion of the experiments based on the use of guide cannulae, animals were killed by deeply anesthesia with an intraperitoneal injection of sodium pentobarbital (45 mg/kg body weight) and probe localization was verified after brain removal. Only animals with correct localizations were considered for further analysis.

**Statistical analyses**

Data were first analyzed using full-factorial parametric One or two-way ANOVA to identify main effects of the manipulations used as well as their interactions (summary in Table 1-2), followed by pre-planned Fisher's LSD or t-tests. A p-value of <0.05 was used for statistical significance. All statistical analyses were performed using the statistical software GraphPad Prism (version 7.0) implemented on a PC/Mac running the Windows 7/MacOS Sierra operating system.

**References**

1. Amato D, Vernon AC, Papaleo F. Dopamine, the antipsychotic molecule: A perspective on mechanisms underlying antipsychotic response variability. *Neuroscience and biobehavioral reviews* 2017.

2. Farde L, Nordstrom AL, Wiesel FA, Pauli S, Halldin C, Sedvall G. Positron emission tomographic analysis of central D1 and D2 dopamine receptor occupancy in patients treated with classical neuroleptics and clozapine. Relation to extrapyramidal side effects. *Archives of general psychiatry* 1992; **49**(7)**:** 538-544.

3. Kapur S, Zipursky R, Jones C, Remington G, Houle S. Relationship between dopamine D(2) occupancy, clinical response, and side effects: a double-blind PET study of first-episode schizophrenia. *The American journal of psychiatry* 2000; **157**(4)**:** 514-520.

4. Kapur S, VanderSpek SC, Brownlee BA, Nobrega JN. Antipsychotic dosing in preclinical models is often unrepresentative of the clinical condition: a suggested solution based on in vivo occupancy. *The Journal of pharmacology and experimental therapeutics* 2003; **305**(2)**:** 625-631.

5. Samaha AN, Seeman P, Stewart J, Rajabi H, Kapur S. "Breakthrough" dopamine supersensitivity during ongoing antipsychotic treatment leads to treatment failure over time. *The Journal of neuroscience : the official journal of the Society for Neuroscience* 2007; **27**(11)**:** 2979-2986.

6. El Hage C, Bedard AM, Samaha AN. Antipsychotic treatment leading to dopamine supersensitivity persistently alters nucleus accumbens function. *Neuropharmacology* 2015; **99:** 715-725.

7. McCormick PN, Kapur S, Graff-Guerrero A, Raymond R, Nobrega JN, Wilson AA. The antipsychotics olanzapine, risperidone, clozapine, and haloperidol are D2-selective ex vivo but not in vitro. *Neuropsychopharmacology : official publication of the American College of Neuropsychopharmacology* 2010; **35**(8)**:** 1826-1835.

8. Amato D, Natesan S, Kapur S, Muller CP. Haloperidol modulates noradrenergic responses to aversive stimulation depending on treatment duration. *Behavioural brain research* 2011; **221**(1)**:** 311-313.

9. Amato D, Natesan S, Yavich L, Kapur S, Muller CP. Dynamic regulation of dopamine and serotonin responses to salient stimuli during chronic haloperidol treatment. *The international journal of neuropsychopharmacology / official scientific journal of the Collegium Internationale Neuropsychopharmacologicum* 2011; **14**(10)**:** 1327-1339.

10. Wadenberg ML, Soliman A, VanderSpek SC, Kapur S. Dopamine D(2) receptor occupancy is a common mechanism underlying animal models of antipsychotics and their clinical effects. *Neuropsychopharmacology : official publication of the American College of Neuropsychopharmacology* 2001; **25**(5)**:** 633-641.

11. Lopez-Nicolas JM, Rodriguez-Bonilla P, Garcia-Carmona F. Cyclodextrins and antioxidants. *Critical reviews in food science and nutrition* 2014; **54**(2)**:** 251-276.

12. Olesen OV, Linnet K. Determination of olanzapine in serum by high-performance liquid chromatography using ultraviolet detection considering the easy oxidability of the compound and the presence of other psychotropic drugs. *Journal of chromatography B, Biomedical sciences and applications* 1998; **714**(2)**:** 309-315.

13. Remington G, Mann S, McCormick P, Nobrega JN, Hahn M, Natesan S. Modeling chronic olanzapine exposure using osmotic minipumps: pharmacological limitations. *Pharmacology, biochemistry, and behavior* 2011; **100**(1)**:** 86-89.

14. Vernon AC, Natesan S, Modo M, Kapur S. Effect of chronic antipsychotic treatment on brain structure: a serial magnetic resonance imaging study with ex vivo and postmortem confirmation. *Biological psychiatry* 2011; **69**(10)**:** 936-944.

15. Tischbirek CH, Wenzel EM, Zheng F, Huth T, Amato D, Trapp S *et al.* Use-dependent inhibition of synaptic transmission by the secretion of intravesicularly accumulated antipsychotic drugs. *Neuron* 2012; **74**(5)**:** 830-844.

16. Camarero J, Sanchez V, O'Shea E, Green AR, Colado MI. Studies, using in vivo microdialysis, on the effect of the dopamine uptake inhibitor GBR 12909 on 3,4-methylenedioxymethamphetamine ('ecstasy')-induced dopamine release and free radical formation in the mouse striatum. *Journal of neurochemistry* 2002; **81**(5)**:** 961-972.

17. Mechan AO, Esteban B, O'Shea E, Elliott JM, Colado MI, Green AR. The pharmacology of the acute hyperthermic response that follows administration of 3,4-methylenedioxymethamphetamine (MDMA, 'ecstasy') to rats. *British journal of pharmacology* 2002; **135**(1)**:** 170-180.

18. Amato D, Muller CP, Badiani A. Increased drinking after intra-striatal injection of the dopamine D2/D3 receptor agonist quinpirole in the rat. *Psychopharmacology* 2012; **223**(4)**:** 457-463.

19. Gulbins E, Palmada M, Reichel M, Luth A, Bohmer C, Amato D *et al.* Acid sphingomyelinase-ceramide system mediates effects of antidepressant drugs. *Nature medicine* 2013; **19**(7)**:** 934-938.

20. C. PGaW. *The rat brain in stereotactic coordinates*. New York: Academic1986.

21. Amato D, Canneva F, Nguyen HP, Bauer P, Riess O, von Horsten S *et al.* Capturing schizophrenia-like prodromal symptoms in a spinocerebellar ataxia-17 transgenic rat. *Journal of psychopharmacology (Oxford, England)* 2016.

22. Urbach YK, Raber KA, Canneva F, Plank AC, Andreasson T, Ponten H *et al.* Automated phenotyping and advanced data mining exemplified in rats transgenic for Huntington's disease. *Journal of neuroscience methods* 2014; **234:** 38-53.

23. Amato D, Pum ME, Groos D, Lauber AC, Huston JP, Carey RJ *et al.* Neuropharmacology of light-induced locomotor activation. *Neuropharmacology* 2015; **95:** 243-251.

24. Heffner TG, Hartman JA, Seiden LS. A rapid method for the regional dissection of the rat brain. *Pharmacology, biochemistry, and behavior* 1980; **13**(3)**:** 453-456.

25. Mukherjee J, Yang ZY, Das MK, Brown T. Fluorinated benzamide neuroleptics--III. Development of (S)-N-[(1-allyl-2-pyrrolidinyl)methyl]-5-(3-[18F]fluoropropyl)-2, 3-dimethoxybenzamide as an improved dopamine D-2 receptor tracer. *Nuclear medicine and biology* 1995; **22**(3)**:** 283-296.

26. Cumming P, Maschauer S, Riss PJ, Tschammer N, Fehler SK, Heinrich MR *et al.* Radiosynthesis and validation of (1)(8)F-FP-CMT, a phenyltropane with superior properties for imaging the dopamine transporter in living brain. *Journal of cerebral blood flow and metabolism : official journal of the International Society of Cerebral Blood Flow and Metabolism* 2014; **34**(7)**:** 1148-1156.

27. la Fougere C, Boning G, Bartmann H, Wangler B, Nowak S, Just T *et al.* Uptake and binding of the serotonin 5-HT1A antagonist [18F]-MPPF in brain of rats: effects of the novel P-glycoprotein inhibitor tariquidar. *NeuroImage* 2010; **49**(2)**:** 1406-1415.

28. Zhou Y, Endres CJ, Brasic JR, Huang SC, Wong DF. Linear regression with spatial constraint to generate parametric images of ligand-receptor dynamic PET studies with a simplified reference tissue model. *NeuroImage* 2003; **18**(4)**:** 975-989.

29. Cumming P, Maschauer S, Riss PJ, Grill E, Pischetsrieder M, Kuwert T *et al.* Perturbed Development of Striatal Dopamine Transporters in Fatty Versus Lean Zucker Rats: a Follow-up Small Animal PET Study. *Molecular imaging and biology : MIB : the official publication of the Academy of Molecular Imaging* 2015; **17**(4)**:** 521-528.

30. Roeper J. Dissecting the diversity of midbrain dopamine neurons. *Trends in neurosciences* 2013; **36**(6)**:** 336-342.

31. Grace AA, Onn SP. Morphology and electrophysiological properties of immunocytochemically identified rat dopamine neurons recorded in vitro. *The Journal of neuroscience : the official journal of the Society for Neuroscience* 1989; **9**(10)**:** 3463-3481.

32. Lacey MG, Mercuri NB, North RA. Two cell types in rat substantia nigra zona compacta distinguished by membrane properties and the actions of dopamine and opioids. *The Journal of neuroscience : the official journal of the Society for Neuroscience* 1989; **9**(4)**:** 1233-1241.

33. Wienisch M, Klingauf J. Vesicular proteins exocytosed and subsequently retrieved by compensatory endocytosis are nonidentical. *Nature neuroscience* 2006; **9**(8)**:** 1019-1027.

34. Wilhelm BG, Groemer TW, Rizzoli SO. The same synaptic vesicles drive active and spontaneous release. *Nature neuroscience* 2010; **13**(12)**:** 1454-1456.

35. Hua Y, Sinha R, Thiel CS, Schmidt R, Huve J, Martens H *et al.* A readily retrievable pool of synaptic vesicles. *Nature neuroscience* 2011; **14**(7)**:** 833-839.

36. Rother M, Brauner JM, Ebert K, Welzel O, Jung J, Bauereiss A *et al.* Dynamic properties of the alkaline vesicle population at hippocampal synapses. *PloS one* 2014; **9**(7)**:** e102723.

37. Threadgill R, Bobb K, Ghosh A. Regulation of dendritic growth and remodeling by Rho, Rac, and Cdc42. *Neuron* 1997; **19**(3)**:** 625-634.

38. Sankaranarayanan S, De Angelis D, Rothman JE, Ryan TA. The use of pHluorins for optical measurements of presynaptic activity. *Biophysical journal* 2000; **79**(4)**:** 2199-2208.

39. Welzel O, Henkel AW, Stroebel AM, Jung J, Tischbirek CH, Ebert K *et al.* Systematic heterogeneity of fractional vesicle pool sizes and release rates of hippocampal synapses. *Biophysical journal* 2011; **100**(3)**:** 593-601.

40. Jung J, Loy K, Schilling EM, Rother M, Brauner JM, Huth T *et al.* The antidepressant fluoxetine mobilizes vesicles to the recycling pool of rat hippocampal synapses during high activity. *Molecular neurobiology* 2014; **49**(2)**:** 916-930.

**Legend Figures:**

Figure S1. Effects of HAL or OLA on the tyrosine hydroxylase (TH) protein expression in the caudate-putamen (CPu), nucleus accumbens (NAcc) and prefrontal cortex (PFC)**.** (**a**) Haloperidol (HAL) treatment for 14 days increased the TH expression in the CPu compared to control (day 0) (p=0.0376). No other effects were detected. (**b**) HAL did not change TH expression in the NAcc. (**c**) HAL treatment for 2 days increased the TH expression in the PFC compared to control (day 0) (p=0.009). No other effects were detected. N=8/group. (**d**) Olanzapine (OLA) treatment for 14 days increased the TH expression in the CPu compared to control (day 0) (p=0.0453) and compared to OLA 6 days (p=0.0038). No OLA effects on TH were detected after 6 days nor the OLA treatment lead to TH expression changes in the NAcc (**e**) or PFC (**f**) at any time points. N=6-8/group. All data are means + S.D. (*p<0.05, **p<0.01). Statistical significance represents post hoc comparison.

Figure S2. Changes in dopamine transporter (DAT) expression and in the inter-individual response to treatment in the caudate-putamen (CPu), nucleus accumbens (NAcc) and prefrontal cortex (PFC) after 0, 6 and 14 continuous treatment with Olanzapine (OLA). (**a**) OLA did not change the mean group DAT expression in the CPu at any treatment time points compared to control (day 0), but OLA 14 days has increased DAT compared to OLA 6 days (p=0.0319). (**b**) OLA increased the inter-individual responses in the CPu, expressed as the mean absolute deviation (MAD = data value – group mean) after 14 days treatment compared to control (day 0) (p=0.0158) and compared to OLA 6 days (p=0.0279). (**c-f**) OLA did not change the mean group DAT expression in NAcc and in PFC nor it has changed the inter-individual responses to treatment. All data are means + S.D. (*p<0.05). N=6-8/group. Statistical significance represents post hoc comparison.

Figure S3. Changes in dopamine transporter (DAT) expression and in the inter-individual response to treatment in the caudate-putamen (CPu), nucleus accumbens (NAcc) and prefrontal cortex (PFC) after 0, 2, 6 and 14 continuous treatment with haloperidol (HAL). (**a**) HAL did not change the mean group DAT expression in the CPu, (**b**) but it has increased the inter-individual responses, expressed as the mean absolute deviation (MAD), after 14 days treatment compared to control (day 0) (p=0.0004) and compared to HAL 2 days (p=0.0158). (**c**) HAL did not change the mean group DAT expression in the NAcc, (**d)** but it has increased the inter-individual responses after 14 days treatment compared to control (day 0) (p=0.049). (**e**) HAL did not change the mean group DAT expression in the PFC, (**f**) but it has increased the inter-individual responses after 14 days treatment compared to HAL 2 days (p=0.0197). All data are means + S.D. (*p<0.05, **p<0.01, ***p<0.001). N=8/group. Statistical significance represents post hoc comparison.

Figure S4. Changes in noradrenaline transporter (NET) expression and in the inter-individual response to treatment in the caudate-putamen (CPu), nucleus accumbens (NAcc) and prefrontal cortex (PFC) after 0, 2, 6 and 14 continuous treatment with haloperidol (HAL).

(**a**) HAL decreased the mean group NET expression in the CPu after 14 days treatment compared to HAL 2 days (p=0.0429), (**b**) but it has not changed the inter-individual responses, expressed as the mean absolute deviation (MAD). (**c**) HAL 14 days decreased the mean group NET expression in the NAcc after 14 days treatment compared to control (day 0) (p=0.0243), HAL 2 (p=0.0018) and HAL 6 days treatment (p=0.0112). (**d**) HAL has also changed the inter-individual responses in the NAcc after 14 days treatment compared to HAL 2 days (p=0.0341). (**e**) HAL decreased the mean group NET expression in the PFC after 14 days treatment compared to 2 (p=0.0085) and 6 (p=0.0071) days, (**f**) but it has not changed the inter-individual responses. All data are means + S.D. (*p<0.05, **p<0.01). N=8/group. Statistical significance represents post hoc comparison.

Figure S5. Changes in serotonin transporter (SERT) and in the inter-individual response to treatment in the caudate-putamen (CPu), nucleus accumbens (NAcc) and prefrontal cortex (PFC) after 0, 2, 6 and 14 continuous treatment with haloperidol (HAL).

(**a-f**) HAL did not change the mean group NET expression in any brain areas nor it has changed the inter-individual responses of animals. All data are means + S.D. N=8/group. Statistical significance represents post hoc comparison.

Figure S6. Relationship between dopamine transporter (DAT) and tyrosine hydroxylase (TH) protein expression in the caudate-putamen (CPu) as a function of the continuous treatment with haloperidol (HAL), olanzapine (OLA) or vehicle (veh). (**a-d**) The expression of DAT and TH is regulated independently under the treatment with veh, HAL 2 days or with OLA 6 days. N=8/group.

Figure S7. Haloperidol **(**HAL) effect on TP-induced locomotion. HAL 14 days treatment fails to inhibit tail pinch (TP)-induced hyperlocomotion. However, HAL decreased basal locomotion level compared to vehicle (veh) (p<0.01, main effect). N=4-9/group. Data are means + S.E.M *p<0.05, **p<0.01, ***p<0.001, ****p<0.0001. Significance are post hoc comparison when not specified.

**Table S1**

**Statistic summary main figures**

| **Test** | **Dependent measure** | **ANOVA** | **Effects** | **DF** | **F-Value** | **P-Value** | **Figure** |
| --- | --- | --- | --- | --- | --- | --- | --- |
| AMPH-induced  Locomotion | | 2 x 12  (HAL6d  x  AMPH) | Treatment  Time (RM)  Interaction | (1, 144)  (11, 132)  (11, 144) | 57.32  8.148  1.626 | **<0.0001**  **<0.0001**  0.0972 | 1a |
|  |  | 2 x 12  (HAL14d  x  AMPH) | Treatment  Time (RM)  Interaction | (1, 144)  (11, 132)  (11, 144) | 0.1936  20.08  1.551 | 0.6606  **<0.0001**  0.1197 | 1b |
|  |  | 2 x 12  (OLA 2d  x  AMPH) | Treatment  Time (RM)  Interaction | (1, 84)  (11, 77)  (11, 84) | 85.79  25.37  0.9197 | **<0.0001**  **<0.0001**  0.5150 | 1c |
|  |  | 2 x 12  (OLA 6d  x  AMPH) | Treatment  Time (RM)  Interaction | (1, 6)  (11, 66)  (11, 66) | 0.8576  21.87  0.1242 | 0.3901  **<0.0001**  0.9997 | 1d |
| AMPH-induced  PPI deficit | PPI (start-reflex) | Treatment  (2 days) | Treatment | (3, 28) | 2.344 | 0.0944 | 1e |
|  | PPI (start-reflex) | Treatment  (14 days) | Treatment | (3, 28) | 15.3 | P<0.0001 | 1f |
| TP-induced locomotion | Locomotion | 3 x 4  (HAL  x  Time) | Treatment  Time (RM)  Interaction | (2, 138)  (3, 105)  (6, 138) | 7.621  33.12  1.414 | **0.0007**  **<0.0001**  0.2136 | 1g |
| TP-induced  locomotion | Locomotion | 4 x 5  (OLA  x  Time) | Treatment  Time (RM)  Interaction | (3,228)  (5,190)  (15,228) | 2.498  11.99  0.4839 | 0.0605  **<0.0001**  0.9473 | 1h |
| D2/3 receptor occupancy during haloperidol failure | D2/3 receptor occupancy | t-test | Treatment | (6) | t=8.728 | 0.0001 | 2a |
| Microdialysis: Antipsychotic effects on dopamine basal  levels | DA levels  (pg/20min, CPU) | OLA  (0, 2, 6, 14 days) | Treatment day | (3,25) | 6.928 | **0.0015** | 2b |
|  | DA levels (pg/20min, NAcc) | OLA  (0, 2, 6, 14 days) | Treatment day | (3,24) | 4.017 | **0.0189** | 2c |
|  | DA levels  (pg/20min, PFC) | OLA  (0, 2, 6, 14 days) | Treatment day | (3,19) | 2.397 | 0.1000 | 2d |
|  | DA levels  (pg/20min, CPU) | HAL  (0, 6, 14 days) | Treatment day | (2,12) | 6.706 | **0.0111** | 2e |
|  | DA levels (pg/20min, NAcc) | HAL  (0, 6, 14 days) | Treatment day | (2,11) | 1.430 | 0.2806 | 2f |
|  | DA levels (pg/20min, NAcc) | t-test  (0 vs. 6 days) | Treatment day | (7) | t=3.382 | **0.0059** | 2f |
|  | DA levels (pg/20min, NAcc) | t-test  (0 vs. 14 days) | Treatment day | (8) | t=0.464 | 0.3273 | 2f |
|  | DA levels  (pg/20min, PFC) | HAL  (0, 6, 14 days) | Treatment day | (2,8) | 6.376 | **0.0221** | 2g |
| Relationship  TP-induced dopamine output and DAT expression | Correlation  DA levels (CPu) - DA (%change) | Pearson r | Treatment (HAL14d) | - | -0.5512 | **0.0394** | 2h |
|  |  |  | Treatment (OLA21d) | - | -0.9298 | **0.0110** | 2i |
| Relationship  TH and DAT expression | Correlation  TH-DAT levels (CPu) |  | Treatment (HAL6d) | - | 0.7438 | **0.0172** | 3a |
|  |  |  | Treatment (HAL14d) | - | 0.9356 | **0.0003** | 3b |
|  |  |  | Treatment (OLA14d) | - | 0.8107 | **0.0073** | 3c |
|  |  |  | Treatment  (veh) | - | 0.1542 | 0.3853 | 3d |
| Haloperidol treatment effect on DAT mRNA expression | DAT mRNA expression (Substantia nigra) | HAL  (0, 2, 6, 14 days) | Treatment day | (3, 26) | 3.716 | **0.0239** | 3e |
| Haloperidol treatment effect on DAT mRNA expression | DAT mRNA expression (Ventral Tegmental Area) | HAL  (0, 2, 6, 14 days) | Treatment day | (3,26) | 2.768 | 0.0618 | 3f |
| Haloperidol effect on dopamine transporter protein density in vivo  and its relationship with TP-induced locomotion | DAT density | t-test | HAL14  (Day 0-14) | (15) | t=4.690 | **0.0001** | 3g-h |
|  | Correlation  DAT_BP_ levels - TP-indiced locomotion | Spearman r | Treatment (HAL14d) | - | 0.7167 | **0.0184** | 3i |
|  | Comparison  DAT_BP_ levels – Antipsychotic efficacy | t-test | HAL14  (Efficacy vs. Failure prone) | (7) | 3.615 | **0.0043** | 3j |

| Test | Dependent measure | Statistical test | Effects | Stat.  value | Stat.  value | p-value | Figure |
| --- | --- | --- | --- | --- | --- | --- | --- |
| Haloperidol effect on spontaneously active SNc DA neurons | Action potential frequency | One-tailed  t-test | Treatment | Means  0.878/1.553  (14D vsVeh)  1.5375/1.553  (6DvsVeh) | t=1.676  t=0.039 | 0.05    0.97 | 4c |
| Haloperidol effect on spontaneously inactive SNc DA neurons | resting membrane potential | One-tailed  t-test | Treatment | Means  -53.75/-48.44  (14D vsVeh)  -55.11/-48.44  (6DvsVeh) | t=1.83  t=2.525 | <0.05  <0.013 | 4d |
| Haloperidol effect on synaptic transmission in CPu | Absolute synaptic field potential amplitude | Two-way ANOVA | Treatment  Time  Interaction | DF  2  7  14 | F-value  5.215  309.7  1.507 | 0.0122  <0.0001  0.1121 | 4f |
| CPu synaptic responses during 40 stimuli at 25 Hz | Relative synaptic field potential amplitude | Kruskal-Wallis | Treatment | KW Stat  9.387  (p=0.0092) | Diff rank sum  -3.463 (6DvsVeh)  -22.15 (14DvsVeh)  -18.69 (6Dvs14D) | Ns  <0.05  <0.05 | 4g |
| Haloperidol effect on isolated axonal activity | Relative fiber volley amplitude | Kruskal-Wallis | Treatment | KW Stat  18.94  (p<0.0001) | Diff rank sum  32.56 (6DvsVeh)  24.29 (14DvsVeh)  -8.275 (6Dvs14D) | <0.001  <0.01  Ns | 4h |

| **Test** | | **dependent measure** | **t-test** | **DF** | **F-Value** | **p-Value** | **Figure** |
| --- | --- | --- | --- | --- | --- | --- | --- |
| Haloperidol effect | Optogenetic  measurement of synaptic vesicle pool sizes | recycling pool size  [RP, % total pool size] | 1h: DMSO vs. HAL | (20) | 0.97 | 0.3415 | 5f |
|  |  |  | 6d: DMSO vs. HAL | (24) | 2.88 | **0.0083** | 5f |
|  |  |  | 14d: DMSO vs. HAL | (34) | -0.89 | 0.3796 | 5f |
|  |  |  | DMSO: 1h vs. 6d | (21) | -0.30 | 0.7659 | 5f |
|  |  |  | DMSO: 1h vs. 14d | (28) | 1.02 | 0.3156 | 5f |
|  |  |  | DMSO: 6d vs. 14d | (33) | 1.39 | 0.1746 | 5f |
|  |  |  | HAL: 1h vs. 6d | (23) | 1.94 | 0.0649 | 5f |
|  |  |  | HAL: 1h vs. 14d | (26) | -0.76 | 0.4563 | 5f |
|  |  |  | HAL: 6d vs. 14d | (25) | -2.43 | **0.0227** | 5f |
|  |  | readily releasable pool size  [RRP, % total pool size] | 1h: DMSO vs. HAL | (20) | 0.94 | 0.3563 | 5g |
|  |  |  | 6d: DMSO vs. HAL | (24) | 0.41 | 0.6852 | 5g |
|  |  |  | 14d: DMSO vs. HAL | (34) | -1.85 | 0.0729 | 5g |
|  |  |  | DMSO: 1h vs. 6d | (21) | 0.36 | 0.7220 | 5g |
|  |  |  | DMSO: 1h vs. 14d | (28) | 2.11 | **0.0444** | 5g |
|  |  |  | DMSO: 6d vs. 14d | (33) | 1.61 | 0.1166 | 5g |
|  |  |  | HAL: 1h vs. 6d | (23) | 0.13 | 0.8986 | 5g |
|  |  |  | HAL: 1h vs. 14d | (26) | -0.49 | 0.6316 | 5g |
|  |  |  | HAL: 6d vs. 14d | (25) | -0.46 | 0.6463 | 5g |

| **Test** | | **Dependent measure** | **ANOVA** | **Effects** | **DF** | **F-Value** | **P-Value** | **Figure** |
| --- | --- | --- | --- | --- | --- | --- | --- | --- |
| Haloperidol Efficacy | K^+^-induced  dopamine release | Dopamine release  (CPU) | 3 x 5  (Veh, HAL6, HAL14  x K^+^) | Treatment  Time (RM)  Interaction | (2,60)  (4,48)  (8,60) | 1.089  10.25  0.774 | 0.3432  **<0.0001**  0.6271 | 5h |
|  |  | Dopamine release  (NAcc) | 3 x 5  (Veh, HAL6, HAL14  x K^+^) | Treatment  Time (RM)  Interaction | (2,55)  (4,44)  (8,44) | 0.7639  2.333  0.4723 | 0.4707  0.0704  0.8704 | 5i |
|  | K^+^-induced  locomotion | Locomotion | 3 x 5  (Veh, HAL6, HAL14  x  K^+^) | Treatment  Time (RM)  Interaction | (2,95)  (4,76)  (8,95) | 0.864  3.307  0.632 | 0.4247  **0.0149**  0.7491 | 5j |

| **Test** | | **Dependent measure** | **ANOVA/t-test**  **(two tailed)** | **Effects** | **DF** | **F-Value** | **P-Value** | **Figure** |
| --- | --- | --- | --- | --- | --- | --- | --- | --- |
| Haloperidol Efficacy | Microdialysis  Basal DA | DA  (pg/20min, CPU) | 2 x 3  (Veh x HAL14) | Treatment  Time (RM)  Interaction | (1,36)  (2,24)  (2,36) | 4.186  2.079  0.0468 | **0.0481**  0.1470  0.9543 | 6a |
|  | TP-induced locomotion | Locomotion | 2 x 4  (Veh/HAL14 x TP) | Treatment  Time (RM)  Interaction | (1,48)  (3,36)  (3,48) | 2.489  26.99  0.623 | 0.1212  **<0.0001**  0.6034 | 6b |

| **Test** | | **Dependent measure** | **ANOVA/t-test**  **(two tailed)** | **Effects** | **DF** | **F-Value** | **P-Value** | **Figure** |
| --- | --- | --- | --- | --- | --- | --- | --- | --- |
| Haloperidol Efficacy:  effect of systemic and intra-caudate GBR12909 | Microdialysis  Basal DA | Basal DA  (pg/20min, CPU) | 2 x 3  (Veh x HAL14 before GBR12909) | Treatment  Time (RM)  Interaction | (1,24)  (2,16)  (2,24) | 4.717  1.424  0.4426 | **0.0400**  0.2696  0.6475 | 6c |
|  |  | Basal DA  (pg/20min, CPU) | 2 x 3  (Veh x HAL14 after GBR12909) | Treatment  Time (RM)  Interaction | (1,24)  (2,16)  (2,24) | 0.5162.107  0.094 | 0.4795  0.1541  0.9102 | 6d |
|  | TP-induced  locomotion | Locomotion | 2 x 4  (Veh/H14+ GBR12909 x TP) | Treatment  Time (RM)  Interaction | (1,32)  (3,24)  (3,32) | 30.17  13.21  1.474 | **<0.0001**  **<0.0001**  0.2401 | 6e |
|  |  |  | Locomotion reduction  (HAL14 vs. HAL14+GBR12909) | Inhibition efficacy | (10) | 2.032 | **0.0348** | 6f |
|  |  |  | 2 x 4  (HAL14 sham-ic/HAL14+ GBR12909-ic x TP) | Treatment  Time (RM)  Interaction | (1,24)  (3,18)  (3,24) | 4.947  1.259  1.746 | **0.0358**  0.3180  0.1844 | 6g |

**Table S2**

**Statistic summary supplementary figures**

| **Test** | | **Dependent measure** | **t-test**  **(one tail)/ANOVA** | **Effects** | **DF** | **t-/F-Value** | **P-Value** | **Figure** |
| --- | --- | --- | --- | --- | --- | --- | --- | --- |
| Haloperidol effect on the tyrosine hydroxilase expression | Western Blot  TH protein expression | TH protein expression  (CPu) | HAL  (0, 2 days) | TH expression change | (14) | 1.142 | 0.1363 | S1a |
|  |  |  | HAL  (0, 6 days) | TH expression change | (14) | 0.3475 | 0.3667 | S1a |
|  |  |  | HAL  (0, 14 days) | TH expression change | (14) | 1.922 | **0.0376** | S1a |
|  |  | TH protein expression  (NAcc) | HAL  (0, 2 days) | TH expression change | (14) | 0.09509 | 0.4628 | S1b |
|  |  |  | HAL  (0, 6 days) | TH expression change | (14) | 0.1113 | 0.4565 | S1b |
|  |  |  | HAL  (0, 14 days) | TH expression change | (14) | 0.7146 | 0.2433 | S1b |
|  |  | TH protein expression  (PFC) | HAL  (0, 2 days) | TH expression change | (14) | 2.679 | **0.0090** | S1c |
|  |  |  | HAL  (0, 6 days) | TH expression change | (14) | 0.5460 | 0.2968 | S1c |
|  |  |  | HAL  (0, 14 days) | TH expression change | (14) | 0.6657 | 0.2582 | S1c |
|  |  | TH protein expression  (CPu) | OLA  (0, 6, 14 days) | TH expression change | (2, 19) | 5.665 | **0.0118** | S1d |
|  |  | TH protein expression  (NAcc) | OLA  (0, 6 days) | TH expression change | (12) | 0.2320 | 0.4102 | S1e |
|  |  |  | OLA  (0, 14 days) | TH expression change | (12) | 0.9605 | 0.1779 | S1e |
|  |  | TH protein expression  (PFC) | OLA  (0, 6 days) | TH expression change | (11) | 0.08078 | 0.4685 | S1f |
|  |  |  | OLA  (0, 14 days) | TH expression change | (12) | 0.3419 | 0.3692 | S1f |

| **Test** | | **Dependent measure** | **ANOVA** | **Effects** | **DF** | **F-Value** | **P-Value** | **Figure** |
| --- | --- | --- | --- | --- | --- | --- | --- | --- |
| Olanzapine effect on dopamine transporter protein expression | Western Blot  DAT protein expression | DAT  protein expression  (CPu) | OLA  (0, 6, 14 days) | DAT density change | (2,19) | 2.946 | 0.0768 | S2a |
|  |  |  | OLA  (0, 6, 14 days) | DAT Mean absolute deviation | (2,19) | 1.347 | **0.0277** | S2b |
|  |  | DAT  protein expression  (NAcc) | OLA  (0, 6, 14 days) | DAT density change | (2,19) | 0.8332 | 0.4499 | S2c |
|  |  |  | OLA  (0, 6, 14 days) | DAT Mean absolute deviation | (2,19) | 1.370 | 0.2781 | S2d |
|  |  | DAT  protein expression  (PFC) | OLA  (0, 6, 14 days) | DAT density change | (2,19) | 0.8071 | 0.4609 | S2e |
|  |  |  | OLA  (0, 6, 14 days) | DAT Mean absolute deviation | (2,19) | 0.3388 | 0.7169 | S2f |

| **Test** | | **Dependent measure** | **ANOVA** | **Effects** | **DF** | **F-Value** | **P-Value** | **Figure** |
| --- | --- | --- | --- | --- | --- | --- | --- | --- |
| Haloperidol effect on the monoamine transporter protein expression | Western Blot  DAT protein expression | DAT protein expression  (CPu) | HAL  (0, 2, 6, 14 days) | DAT density change | (3,28) | 1.074 | 0.3758 | S3a |
|  |  |  | HAL  (0, 2, 6, 14 days) | DAT Mean absolute deviation | (3,28) | 5.553 | **0.0040** | S3b |
|  |  | DAT protein expression  (NAcc) | HAL  (0, 2, 6, 14 days) | DAT density change | (3,28) | 0.3051 | 0.8215 | S3c |
|  |  |  | HAL  (0, 2, 6, 14 days) | DAT Mean absolute deviation | (3,28) | 1.430 | 0.2550 | S3d |
|  |  | DAT protein expression  (PFC) | HAL  (0, 2, 6, 14 days) | DAT density change | (3,27) | 1.359 | 0.2764 | S3e |
|  |  |  | HAL  (0, 2, 6, 14 days) | DAT Mean absolute deviation | (3,27) | 2.119 | 0.1212 | S3f |
|  | Western Blot  NET  protein expression | NET protein expression  (CPu) | HAL  (0, 2, 6, 14 days) | NET density change | (3,28) | 1.831 | 0.1645 | S4a |
|  |  |  | HAL  (0, 2, 6, 14 days) | NET Mean absolute deviation | (3,28) | 1.168 | 0.3395 | S4b |
|  |  | NET protein expression  (NAcc) | HAL  (0, 2, 6, 14 days) | NET density change | (3,28) | 4.438 | **0.0113** | S4c |
|  |  |  | HAL  (0, 2, 6, 14 days) | NET Mean absolute deviation | (3,28) | 1.903 | 0.1521 | S4d |
|  |  | NET protein expression  (PFC) | HAL  (0, 2, 6, 14 days) | NET density change | (3,28) | 3.702 | **0.0232** | S4e |
|  |  |  | HAL  (0, 2, 6, 14 days) | NET Mean absolute deviation | (3,28) | 0.0938 | 0.9629 | S4f |
|  | Western Blot  SERT  protein expression | SERT protein expression  (CPu) | HAL  (0, 2, 6, 14 days) | SERT density change | (3,27) | 0.655 | 0.5866 | S5a |
|  |  |  | HAL  (0, 2, 6, 14 days) | SERT Mean absolute deviation | (3,27) | 0.735 | 0.5403 | S5b |
|  |  | SERT protein expression  (NAcc) | HAL  (0, 2, 6, 14 days) | SERT density change | (3,26) | 0.4581 | 0.7139 | S5c |
|  |  |  | HAL  (0, 2, 6, 14 days) | SERT Mean absolute deviation | (3,25) | 1.072 | 0.3790 | S5d |
|  |  | SERT protein expression  (PFC) | HAL  (0, 2, 6, 14 days) | SERT density change | (3,17) | 1.490 | 0.253 | S5e |
|  |  |  | HAL  (0, 2, 6, 14 days) | SERT Mean absolute deviation | (3,17) | 1.209 | 0.336 | S5f |

| **Test** | **Dependent measure** | **ANOVA** | **Effects** | **DF** | **F-Value** | **P-Value** | **Figure** |
| --- | --- | --- | --- | --- | --- | --- | --- |
| Relationship between  TH and DAT expression proteins after antipsychotic treatment | veh effect on DAT-TH in CPu | Pearson r | Correlation  (one tail) | - | -0.5199 | 0.0933 | S6a |
|  | HAL 2 days effect on DAT-TH in Cpu | Pearson r | Correlation  (one tail) | - | 0.3734 | 0.1811 | S6b |
|  | OLA 6 days effect on DAT-TH in Cpu | Pearson r | Correlation  (one tail) | - | -0.4756 | 0.1168 | S6d |

| **Test** | | **Dependent measure** | **ANOVA** | **Effects** | **DF** | **F-Value** | **P-Value** | **Figure** |
| --- | --- | --- | --- | --- | --- | --- | --- | --- |
| Haloperidol Efficacy | Behavior  TP-induced locomotion | Locomotion | 2 x 10  (Veh x HAL14) | Treatment  Time (RM)  Interaction | (1,110)  (9,99)  (9,110) | 8.937  4.920  0.9545 | **0.0034**  **<0.0001**  0.4817 | S7 |
